# Unique equilibrium in contests with incomplete information

**DOI:** 10.1007/s00199-019-01209-4

**Published:** 2019-07-09

**Authors:** Christian Ewerhart, Federico Quartieri

**Affiliations:** 1 Department of Economics, University of Zurich, Schönberggasse 1, 8001 Zurich, Switzerland; 2 Department of Economics and Management, University of Florence, Florence, Italy

**Keywords:** Imperfectly discriminating contests, Private information, Existence and uniqueness of equilibrium, Budget constraints, Rent dissipation, C72, D23, D72, D82

## Abstract

Considered are imperfectly discriminating contests in which players may possess private information about the primitives of the game, such as the contest technology, valuations of the prize, cost functions, and budget constraints. We find general conditions under which a given contest of incomplete information admits a unique pure-strategy Nash equilibrium. In particular, provided that all players have positive budgets in all states of the world, existence requires only the usual concavity and convexity assumptions. Information structures that satisfy our conditions for uniqueness include independent private valuations, correlated private values, pure common values, and examples of interdependent valuations. The results allow dealing with inactive types, asymmetric equilibria, population uncertainty, and the possibility of resale. It is also shown that any player that is active with positive probability ends up with a positive net rent.

## Introduction

Contest theory has emerged from the study of important economic problems in areas such as marketing, patent races, R&D, promotion tournaments, political campaigning, legal disputes, lobbying, sports, and military conflict.^1^ As one of its main objectives, the literature has sought to characterize the extent of rent dissipation, i.e., the share of the contested rent that is spent by the competing parties in an attempt to win the contest. Of some value, however, is typically also the assurance that a pure-strategy Nash equilibrium exists and is unique. In particular, the existence of a unique equilibrium may be useful for reasons such as analytical convenience, predictive power, comparative statics, and global stability.

This paper offers general conditions sufficient for the existence and uniqueness of pure-strategy and mixed-strategy Nash equilibria in incomplete-information contests over arbitrary finite state spaces. In the class of contests considered, players may possess private information about the primitives of the conflict, i.e., about the contest technology, valuations, cost functions, and budget constraints. Our assumptions directly generalize the conditions formulated by Szidarovszky and Okuguchi (1997). Thus, we deal with contests of the logit form, for which Tullock’s (1980) rent-seeking game is an important example.

Regarding existence, we show that a pure-strategy Nash equilibrium exists in a large class of incomplete-information contests, provided that no player ever deems it possible being the only one with a positive budget in a state where the technology of success is discontinuous. This result is obtained through the consideration of a sequence of contests with smaller and smaller minimum bids. Indeed, under the assumptions that will be imposed, any discontinuity at the origin does not matter, essentially because small positive bids in a contest of the logit form create very strong incentives to overbid. Our conditions for existence can be further relaxed in certain circumstances, e.g., when the slope of the state-dependent impact function is infinite at the zero bid level, or when the contest is symmetric. However, we also present an example of a contest with incomplete information that does not satisfy our assumptions and that does not admit any equilibrium (even though all posterior beliefs assign positive probability to the possibility that the respective opponent has a positive budget).^2^

Regarding uniqueness, our contribution has four main elements. First, we extend Rosen’s (1965) notion of “diagonal strict concavity” to a class of *n*-player logit contests. The extension is needed because, even in a standard Tullock contest, payoff functions are neither continuous nor strictly concave (at the boundary).^3^ Second, we show that, under standard assumptions, any *n*-player logit contest satisfies the sufficient conditions of Goodman (1980) in the interior of the set of strategy profiles.^4^ Third, we identify a simple assumption on the information structure that ensures that the uniqueness proof goes through. Thereby, we can deal not only with independent private valuations, correlated private values, and pure common values, but also with examples of interdependent valuations. Finally, we show that the pure-strategy Nash equilibrium remains unique in the larger set of mixed-strategy Nash equilibria.

Several papers have stressed the role of budget constraints for the outcome of contests.^5^ Our framework accounts for this possibility by allowing for type-dependent budget constraints. This creates a variety of modeling choices. For example, under the assumption of convex and strictly increasing cost functions, a budget may be so generous that it never binds. At the opposite extreme, any type with a zero budget will be effectively excluded from being active in the contest. This latter possibility may then be used, for example, to deal with the case with population uncertainty.

The remainder of this paper is structured as follows. The related literature is discussed in Sect. 2. Section 3 introduces the setup and notation. Existence is dealt with in Sect. 4, while Sect. 5 discusses uniqueness. Section 6 considers symmetric contests. Applications are provided in Sect. 7. Section 8 concludes. All proofs have been relegated to an Appendix.

## Related literature

The existence and uniqueness of a pure-strategy Nash equilibrium in imperfectly discriminating contests have been studied quite thoroughly in the case of complete information (Pérez-Castrillo and Verdier 1992; Baye et al. 1993; Szidarovszky and Okuguchi 1997; Esteban and Ray 1999; Cornes and Hartley 2005, 2012; Yamazaki 2008, 2009; Franke and Öztürk 2015), and in the case of symmetrically informed contestants (Einy et al. 2017). For contests with incomplete information, however, be it with one-sided incomplete information and continuous types (Hurley and Shogren 1998a), discrete type spaces (Hurley and Shogren 1998b; Malueg and Yates 2004; Schoonbeek and Winkel 2006), a continuously distributed common valuation (Harstad 1995; Wärneryd 2003, 2012; Rentschler 2009), continuously and independently distributed marginal costs (Fey 2008; Ryvkin 2010; Wasser 2013a), or continuously distributed interdependent valuations (Wasser 2013b), the results have been overall somewhat less comprehensive.

There has been fairly little work especially on the issue of uniqueness of an equilibrium in a contest with incomplete information. A notable exception is the seminal paper on contests with two-sided incomplete information, Hurley and Shogren (1998b). Considering a private valuations framework with two players, where one player has two types and the other player has three types, they have shown that there is at most one interior equilibrium. While interesting, their application of the index theorem is actually quite involved, and it is not obvious that the approach could be generalized. Even if a generalization was feasible, boundary equilibria would remain a possibility. Another notable exception is Wasser (2013a) who proves uniqueness in a class of IPV contests with continuous and strictly concave payoff functions. However, that result requires parametric assumptions.^6^

Although some results of the present analysis extend to continuous, independently distributed types (first-named author 2014), our focus on finite state spaces leads to additional flexibility in other dimensions. Specifically, in the analysis below, private information may concern not only a marginal cost parameter but also the contest technology, valuations, the shape of cost functions, and budget constraints. Moreover, the present analysis is not restricted to the IPV framework, but allows for a very large variety of alternative information structures. Of course, dealing with these possibilities leads, in particular, to additional complications in the proofs.

Equilibrium existence in a state-space framework has been studied also by Einy et al. (2015). Their main result, derived through an application of Reny’s theorem, is complementary to Theorem 1 in that their conditions are consistent with countably infinite information partitions and nonsmooth contest technologies. However, as mentioned before, Einy et al. (2015) do not allow for budget constraints, nor do they offer general conditions for uniqueness.^7^

## Setup and notation

There are $n\ge 2$ contestants, or players, collected in a set $N=\{1,\ldots ,n\}$. All uncertainty about the primitives of the contest is summarized in a state variable $\omega$ that is drawn ex ante from a finite state space $\Omega$. We denote by $q(\omega )$ the ex ante probability of state $\omega$, where we assume $q(\omega )>0$ for all $\omega \in \Omega$.

Just before the contest, each player $i\in N$ receives a signal (or type) $\theta _{i}=t_{i}(\omega )$ from a nonempty signal space $\Theta _{i}$. Signals are private information, i.e., player *i* does not observe the signal $\theta _{j}=t_{j}(\omega )$ received by any other player $j\ne i$. We denote by ${\mathcal {P}}_{i}(\theta _{i})=\{\omega \in \Omega \vert t_{i}(\omega )=\theta _{i}\}$ the set of states deemed possible by type $\theta _{i}$, and assume that ${\mathcal {P}}_{i}(\theta _{i})\ne \varnothing$. Then, clearly, the (unconditional) probability that a given signal $\theta _{i}$ realizes, i.e., $q_{i}(\theta _{i})= {\textstyle \sum _{\omega \in {\mathcal {P}}_{i}(\theta _{i})}} q(\omega )$, is positive for any $i\in N$ and $\theta _{i}\in \Theta _{i}$. Finally, we assume that each type $\theta _{i}\in \Theta _{i}$ of any player $i\in N$ forms a posterior $q_{i}(\cdot \vert \theta _{i})$ on $\Omega$ via Bayes’ rule, so that $q_{i}(\omega \vert \theta _{i})=q(\omega )/q_{i}(\theta _{i})$ if $\omega \in {\mathcal {P}}_{i} (\theta _{i})$, and $q_{i}(\omega \vert \theta _{i})=0$ otherwise.

After observing $\theta _{i}\in \Theta _{i}$, each player $i\in N$ chooses an expenditure level, or bid, $x_{i}=\beta _{i}(\theta _{i})\in [0,x_{i} ^{\max }(\theta _{i})]$, where $x_{i}^{\max }(\theta _{i})\ge 0$ denotes the *budget* of type $\theta _{i}$. It is worthwhile to note that, in contrast to the other primitives of the model, budgets are functions of the type rather than of the state. Note also that assuming a finite budget for all players in all states of the world does not entail any loss of generality provided that (as will be assumed below) cost functions are convex and strictly increasing.^8^ Moreover, as will be illustrated, our assumptions allow for the possibility that $x_{i}^{\max }(\theta _{i})=0$, in which case type $\theta _{i}$ has a zero budget.

Contestant *i*’s probability of winning in state $\omega$ is given by

1 $$\begin{aligned} p_{i,\omega }(x_{1},\ldots ,x_{n})=\left\{ \begin{array}{ll} \dfrac{f_{i,\omega }(x_{i})}{f_{1,\omega }(x_{1})+\cdots +f_{n,\omega }(x_{n})} &{} \text {if }f_{1,\omega }(x_{1})+\cdots +f_{n,\omega }(x_{n})>0\\ &{} \\ p_{i,\omega }^{0} &{} \text {if }f_{1,\omega }(x_{1})+\cdots +f_{n,\omega } (x_{n})=0\text {,} \end{array} \right. \end{aligned}$$

where $f_{i,\omega }(\cdot )\ge 0$ is player *i*’s *impact function* in state $\omega$, and where $p_{i,\omega }^{0}\in [0,1]$ satisfies $p_{1,\omega }^{0}+\cdots +p_{n,\omega }^{0}\le 1$.^9^ For instance, $p_{1,\omega }^{0}=\cdots =p_{n,\omega }^{0}=\frac{1}{n}$ or $p_{1,\omega }^{0}=\cdots =p_{n,\omega }^{0}=0$, as in popular specifications of Tullock’s and other logit contests. For a state $\omega \in \Omega$, we will say that the contest is *discontinuous in*$\omega$ when $f_{1,\omega }(0)+\cdots +f_{n,\omega }(0)=0$. Clearly, $p_{i,\omega }^{0}$ needs to be specified only for states $\omega$ in which the contest is discontinuous. Player *i*’s ex post payoff, or *net rent*, in state $\omega$ is now given by

2 $$\begin{aligned} \Pi _{i,\omega }(x_{1},\ldots ,x_{n})=p_{i,\omega }(x_{1},\ldots ,x_{n})v_{i} (\omega )-c_{i,\omega }(x_{i})\text {,} \end{aligned}$$

where $v_{i}(\omega )>0$ is *i*’s valuation of winning, and $c_{i,\omega } (x_{i})$ is *i*’s cost, both in state $\omega$.

A *bid schedule* for player *i* is a mapping $\beta _{i}:\Theta _{i}\rightarrow {\mathbb {R}} _{+}\equiv [0,\infty )$ such that $\beta _{i}(\theta _{i})\in [0,x_{i}^{\max }(\theta _{i})]$. The set of *i*’s bid schedules is denoted by $B_{i}$. For a given profile of bid schedules $\beta _{-i}=\{\beta _{j}\}_{j\ne i}\in B_{-i}\equiv {\textstyle \prod _{j\ne i}} B_{j}$, denote by $\beta _{-i}(t_{-i}(\omega ))=\{\beta _{j}(t_{j}(\omega ))\}_{j\ne i}$ the profile of bids resulting in state $\omega$, where $t_{-i}(\omega )=\{t_{j}(\omega )\}_{j\ne i}$ is the corresponding profile of type realizations. Similarly, for any $\beta =\{\beta _{i}\}_{i=1}^{n}\in B\equiv {\textstyle \prod _{i=1}^{n}} B_{i}$, we will write $\beta (t(\omega ))=\{\beta _{i}(t_{i}(\omega ))\}_{i=1} ^{n}$, with $t(\omega )=\{t_{i}(\omega )\}_{i=1}^{n}$. The interim expected payoff for type $\theta _{i}\in \Theta _{i}$ of player *i*, when bidding $x_{i}\in [0,x_{i}^{\max }(\theta _{i})]$ against a profile of bid schedules $\beta _{-i}\in B_{-i}$, is given as

3 $$\begin{aligned} {\overline{\Pi }}_{i}(x_{i},\beta _{-i},\theta _{i})= {\sum \limits _{\omega \in {\mathcal {P}}_{i}(\theta _{i})}} q_{i}(\omega \vert \theta _{i})\Pi _{i,\omega }(x_{i},\beta _{-i}(t_{-i}(\omega )))\text {.} \end{aligned}$$

By an *incomplete-information contest *${\mathcal {C}}$, we mean the Bayesian *n*-player game just described. A *pure-strategy Nash equilibrium (PSNE)* in the incomplete-information contest ${\mathcal {C}}$ is a profile of bid schedules $\beta ^{*}=\{\beta _{i}^{*}\}_{i=1}^{n}\in B$ such that ${\overline{\Pi }}_{i}(\beta _{i}^{*}(\theta _{i}),\beta _{-i}^{*},\theta _{i})\ge {\overline{\Pi }}_{i}(x_{i},\beta _{-i}^{*},\theta _{i})$, for any $i\in N$, any $\theta _{i}\in \Theta _{i}$, and any $x_{i}\in [0,x_{i}^{\max }(\theta _{i})]$.

Some of our results concern mixed strategies, where randomization over the continuous bid space is modeled as in Dasgupta and Maskin (1986). Formally, a *mixed strategy*${\mathfrak {b}}_{i}$ for player *i* assigns to each $\theta _{i}\in \Theta _{i}$ a probability measure ${\mathfrak {b}}_{i}(\theta _{i})$ on (the Borel subsets of) the interval $[0,x_{i}^{\max }(\theta _{i})]$. Thus, we assume that types randomize independently. Given a profile of mixed strategies ${\mathfrak {b}}_{-i}=\{{\mathfrak {b}}_{j}\}_{j\ne i}$, type $\theta _{i}$’s expected payoff from a mixed strategy ${\mathfrak {b}}_{i}$ reads

4 $$\begin{aligned} {\overline{\Pi }}_{i}({\mathfrak {b}}_{i}(\theta _{i}),{\mathfrak {b}}_{-i},\theta _{i})= {\sum _{\omega \in {\mathcal {P}}_{i}(\theta _{i})}} q_{i}(\omega \vert \theta _{i})E_{{\mathfrak {b}}(t(\omega ))}\left[ \Pi _{i,\omega }(x)\right] \text {,} \end{aligned}$$

where the expectation $$E_{{\mathfrak {b}}(t(\omega ))}\left[ \cdot \right] $$ at state $$\omega \in {\mathcal {P}}_{i}(\theta _{i})$$ is taken over the realizations of bid profiles $$x=(x_{1},\ldots ,x_{n})$$ according to the product measure $${\mathfrak {b}}(t(\omega ))=({\mathfrak {b}}_{1}(t_{1}(\omega )),\ldots ,$$$${\mathfrak {b}} _{n}(t_{n}(\omega )))$$.^10^ A *mixed-strategy Nash equilibrium (MSNE)* is now a profile $${\mathfrak {b}}^{*}=({\mathfrak {b}}_{1}^{*},\ldots ,{\mathfrak {b}}_{n}^{*})$$ of mixed strategies, one for each player $$i\in N$$, such that $${\overline{\Pi }} _{i}({\mathfrak {b}}_{i}^{*}(\theta _{i}),{\mathfrak {b}}_{-i}^{*},\theta _{i})\ge {\overline{\Pi }}_{i}({\mathfrak {b}}_{i}(\theta _{i}),{\mathfrak {b}} _{-i}^{*},\theta _{i})$$, for any $$i\in N$$ and $$\theta _{i}\in \Theta _{i}$$, and for any mixed strategy $${\mathfrak {b}}_{i}$$ for player *i*. A MSNE $${\mathfrak {b}}^{*}$$ will be called *degenerate* if, for any $$i\in N$$ and $$\theta _{i}\in \Theta _{i}$$, the support of $${\mathfrak {b}}_{i}^{*} (\theta _{i})$$ is a singleton.

## Existence

### Assumptions

We start with the existence part. Two assumptions will be imposed. The first concerns impact and cost functions and generalizes the corresponding assumption in Szidarovszky and Okuguchi (1997) in a straightforward way.

#### Assumption (A)

For any $i\in N$ and $\omega \in \Omega$, the impact function $f_{i,\omega }: {\mathbb {R}} _{+}\rightarrow {\mathbb {R}} _{+}$ is twice differentiable, strictly increasing, and concave. Further, for any $i\in N$ and $\omega \in \Omega$, the cost function $c_{i,\omega }: {\mathbb {R}} _{+}\rightarrow {\mathbb {R}} _{+}$ is twice differentiable, strictly increasing, and convex.

We have two remarks. First, the reader is cautioned that Assumption (A) imposes twice differentiability of impact functions not only in the interior of the strategy space, but also at the zero bid level.^11^ In particular, this point matters for the widely used impact function $f_{i,\omega }(x)=x^{r}$ with $r\in (0,1)$, which is twice differentiable in the interior, but not at the zero bid (where the impact function exhibits a vertical slope). However, in many cases of interest, a simple change of variables, described already in Szidarovszky and Okuguchi (1997), may be used to circumvent the problem. In our setting, this is feasible when each type knows her impact function (i.e., when for any $i\in N$, and any $\omega ,\omega ^{\prime }\in \Omega$ such that $t_{i}(\omega )=t_{i}(\omega ^{\prime })$, we have $f_{i,\omega }(\cdot )=f_{i,\omega ^{\prime }}(\cdot )$). This is particularly easy to see when, in addition, $f_{i,\omega }(0)=0$ for all $i\in N$ and $\omega \in \Omega$.^12^ Then, each player $i\in N$ may be envisaged to choose directly the impact term $y_{i}=f_{i,\omega }(x_{i})\ge 0$ rather than the bid $x_{i}\ge 0$. Since such a change of variables induces a one-to-one transformation of the respective sets of PSNE, and similarly for the respective sets of MSNE, this approach may be employed to expand our results in a useful way and, in particular, to deal with the Tullock example when $r\in (0,1)$.

More formally, one considers a *transformed contest *in which each player $i\in N$, after having received her signal $\theta _{i}=t_{i}(\omega )\in \Theta _{i}$ in state $\omega \in \Omega$, chooses $y_{i}\in [0,y_{i}^{\max }(\theta _{i})]$ at costs ${\widetilde{c}}_{i,\omega }(y_{i} )=c_{i,\omega }(f_{i,\omega }^{-1}(y_{i}))$, where the upper bound of the interval, $y_{i}^{\max }(\theta _{i})=f_{i,\omega }(x_{i}^{\max }(\theta _{i}))$, is well defined as a result of our temporary assumption that each type knows her impact function. Moreover, given a vector $$y=(y_{1},\ldots ,y_{n})\ $$such that $y_{i}\in [0,y_{i}^{\max }(\theta _{i})]$ for all $i\in N$, one specifies any player *i*’s ex post payoff at state $\omega$ as

5 $$\begin{aligned} {\widetilde{\Pi }}_{i,\omega }(y)=\frac{y_{i}}{y_{1}+\cdots +y_{n}}v_{i} (\omega )-{\widetilde{c}}_{i,\omega }(y_{i})\text {,} \end{aligned}$$

where the ratio is interpreted as $$p_{i,\omega }^{0}\ $$if the denominator vanishes. Then, the incomplete-information contest and its transformed counterpart are strategically equivalent in the sense that, for any $i\in N$, any $\omega \in \Omega$, any $x=(x_{1},\ldots ,x_{n})\in {\mathbb {R}} _{+}^{n}$, and any $y=(y_{1},\ldots ,y_{n})\in {\mathbb {R}} _{+}^{n}$ such that $y_{i}=f_{i,\omega }(x_{i})$ for all $i\in N$, we have that (i) $x_{i}\in [0,x_{i}^{\max }(t_{i}(\omega ))]$ if and only if $y_{i} \in [0,y_{i}^{\max }(t_{i}(\omega ))]$, and (ii) ${\widetilde{\Pi }} _{i,\omega }(y)=\Pi _{i,\omega }(x)$. This construction will be illustrated with an example in Sect. 7.

Second, it should be clear that the simple assumptions on concavity and convexity made in Assumption (A) are not crucial for the existence of a PSNE. This point has been noted for contests with complete information (Pérez-Castrillo and Verdier 1992; Nti 1999; Cornes and Hartley 2005) and for contests with incomplete information and discrete types (Malueg and Yates 2004). For continuous types and continuous technologies, convexity assumptions may even be dropped entirely (Wasser 2013b). However, it is also known that, in general, marginal conditions may fail to identify a PSNE in a contest because global second-order conditions need not hold. Assumption (A) excludes this possibility in a straightforward way.

It is fairly easy to see that Assumption (A) alone does not guarantee equilibrium existence. For example, in a standard two-player Tullock contest with complete information and $p_{1}^{0}=p_{2}^{0}=\frac{1}{2}$, there is no equilibrium, neither pure nor mixed, if precisely one player has a positive budget.^13^ Thus, to deal with general cases in which zero budgets may occur in states with discontinuous technologies, we need another assumption.

#### Assumption (B)

For any $i\in N$ and $$\omega \in \Omega \ $$with $x_{i}^{\max }(t_{i}(\omega ))>0$, at least one of the following two conditions holds true:

(i) The contest is continuous in $\omega$;

(ii) There exists $j\in N\backslash \{i\}$ such that $x_{j}^{\max }(t_{j}(\omega ))>0$.

Assumption (B) requires that, in any state of the world in which the contest is discontinuous, there is *not* precisely one contestant with a positive budget. This is a rather mild restriction. For example, it holds when the contest is continuous in all states of the world. Further, the assumption is satisfied when all players have a positive budget in any state of the world.

### Existence result

The first main result of this paper is the following.

#### Theorem 1

Under Assumptions (A) and (B), there exists a PSNE in the incomplete-information contest ${\mathcal {C}}$.

#### Proof

See the Appendix. $\square$

Theorem 1 is proved by means of a simple limit consideration. Specifically, suppose that, for $\varepsilon >0$ small (i.e., smaller than any positive budget), any type with a positive budget is restricted to submitting a bid of at least $\varepsilon$. Then, given Assumption (A), a standard result may be used to establish the existence of a PSNE in the contest with minimum bid. Then, by letting $\varepsilon$ go to zero, a sequence of strategy profiles may be constructed that converges to a PSNE in the unrestricted contest, provided that the limit strategy profile stays clear of the origin in any state of the world in which the contest is discontinuous. But to see that the limit profile cannot be zero in such a state, it suffices to note that, with Assumption (B) in place, the marginal incentive to overbid for *some* endowed player exceeds any finite bound as $\varepsilon \rightarrow 0$. Of course, this conclusion is due to the nature of the discontinuous logit contest that, in terms of marginal incentives, “explodes” for bid vectors that are close to the origin.

### An example of nonexistence

We conclude this section with an example that shows that Assumption (B) cannot be dropped from the statement of Theorem 1 even if budgets are private information.

#### Example 1

**Fig. 1:**
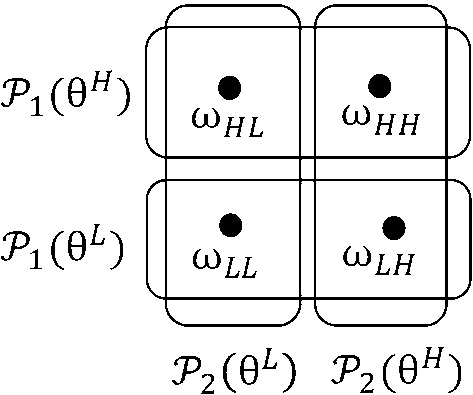
Information structure in Example 1

Consider a two-player *lotterycontest* (i.e., a Tullock contest with parameter $r=1$). Each player has two equally likely types $\theta ^{\text {L}}$ (low) and $\theta ^{\text {H}}$ (high), and type realizations are independent across players. Then, as illustrated in Fig. 1, the state space, $\Omega =\{\omega _{\text {LL}} ,\omega _{\text {LH}},\omega _{\text {HL}},\omega _{\text {HH}}\}$, has four equally likely states, where the first index of the state variable corresponds to player 1’s type (low or high), and the second index to player 2’s type (likewise, low or high). In state $\omega \in \Omega$, player $i\in N=\{1,2\}$ wins with probability

6 $$\begin{aligned} p_{i,\omega }(x_{1},x_{2})=\left\{ \begin{array}{ll} \dfrac{x_{i}}{x_{1}+x_{2}} &{} \text {if }x_{1}+x_{2}>0\\ &{} \\ p_{i,\omega }^{0} &{} \text {if }x_{1}+x_{2}=0\text {,} \end{array} \right. \end{aligned}$$

where $p_{i,\omega }^{0}\in [0,1]$ satisfies $p_{1,\omega }^{0} +p_{2,\omega }^{0}\le 1$. Valuations are given by $v_{1}(\omega _{\text {LL} })=v_{1}(\omega _{\text {LH}})=v_{2}(\omega _{\text {LL}})=v_{2}(\omega _{\text {HL}})=V^{\text {L}}$ and $v_{1}(\omega _{\text {HL}})=v_{1} (\omega _{\text {HH}})=v_{2}(\omega _{\text {LH}})=v_{2}(\omega _{\text {HH} })=V^{\text {H}}$, where $V^{\text {H}}>V^{\text {L}}>0$. Finally, we assume cost functions $c_{i,\omega }(x_{i})=x_{i}$, for any $i\in N$ and $\omega \in \Omega$. This game is known to admit a symmetric interior PSNE with interesting properties (Malueg and Yates 2004; Fey 2008; Ludwig 2012).

We now introduce a budget constraint for player 2’s low type $\theta ^{\text {L}}$ by assuming $x_{2}^{\max }(\theta ^{\text {L}})=0$, while the budgets of the other three types remain sufficiently large to remain irrelevant. It is easy to see that *Assumption* (B)*does not hold.* Indeed, in state $\omega _{\text {LL}}$, the technology is discontinuous while only player 1 has a positive budget. It is claimed now that *there is no PSNE in the parameter domain where *$V^{\text {H}}\ge 4V^{\text {L}}$* and *$p_{1,\omega _{\text {LL}}}^{0}<1$*.* To see this, suppose that an equilibrium is given by bids $x_{1}^{\text {L}}$, $x_{1}^{\text {H}}$, $x_{2}^{\text {L}}$, and $x_{2}^{\text {H}}$, where necessarily $x_{2} ^{\text {L}}=0$. We start by showing that $x_{2}^{\text {H}}$, $x_{1}^{\text {L} }$, and $x_{1}^{\text {H}}$ are all interior, and consequently satisfy the necessary first-order conditions for an interior optimum. Indeed, if we had $x_{2}^{\text {H}}=0$, then type $\theta ^{\text {L}}$ of player 1 would have no best response since $p_{1,\omega _{\text {LL}}}^{0}<1$. Hence, $x_{2}^{\text {H} }>0$, and

7 $$\begin{aligned} \frac{1}{2}\frac{x_{1}^{\text {L}}}{\left( x_{2}^{\text {H}}+x_{1}^{\text {L}}\right) ^{2} }+\frac{1}{2}\frac{x_{1}^{\text {H}}}{\left( x_{2}^{\text {H}}+x_{1}^{\text {H}}\right) ^{2} }=\frac{1}{V^{\text {H}}} \end{aligned}$$

holds true. Next, given $x_{2}^{\text {L}}=0$ and $p_{1,\omega _{\text {LL}}} ^{0}<1$, the best response of player 1’s type $\theta ^{\text {L}}$ must be positive, i.e., we have $x_{1}^{\text {L}}>0$, with

8 $$\begin{aligned} \frac{1}{2}\frac{x_{2}^{\text {H}}}{\left( x_{1}^{\text {L}}+x_{2}^{\text {H}}\right) ^{2} }=\frac{1}{V^{\text {L}}}\text {.} \end{aligned}$$

Finally, since player 1’s best response (if well defined) is monotone increasing in her valuation, one obtains $x_{1}^{\text {H}}>0$, so that

9 $$\begin{aligned} \frac{1}{2}\frac{x_{2}^{\text {H}}}{\left( x_{1}^{\text {H}}+x_{2}^{\text {H}}\right) ^{2} }=\frac{1}{V^{\text {H}}}\text {.} \end{aligned}$$

Solving now (8) and (9) for $x_{1}^{\text {L}}$ and $x_{1}^{\text {H}}$, respectively, yields

10 $$\begin{aligned} x_{1}^{\text {L}}&=\sqrt{\frac{x_{2}^{\text {H}}V^{\text {L}}}{2}} -x_{2}^{\text {H}}\text {, and} \end{aligned}$$

11 $$\begin{aligned} x_{1}^{\text {H}}&=\sqrt{\frac{x_{2}^{\text {H}}V^{\text {H}}}{2}} -x_{2}^{\text {H}}. \end{aligned}$$

Plugging these expressions into (7), and simplifying, we arrive at

12 $$\begin{aligned} x_{2}^{\text {H}}=\frac{V^{\text {L}}}{2}\frac{\left( 1+\sqrt{\dfrac{V^{\text {L}}}{V^{\text {H}}}}\right) ^{2}}{\left( 1+2\dfrac{V^{\text {L}} }{V^{\text {H}}}\right) ^{2}}\text {.} \end{aligned}$$

But from $x_{1}^{\text {L}}>0$ and (10), we get $x_{2}^{\text {H} }<V^{\text {L}}/2$, which is in conflict with (12) if $V^{\text {L} }/V^{\text {H}}\le 1/4$. Thus, unless player 1 wins the prize for sure in state $\omega _{\text {LL}}$ in the case of joint inactivity, there is indeed no PSNE.^14^

Example 1 may be “repaired” by assuming that the endowed player wins with probability one in the case of joint inactivity. Then, in equilibrium, $x_{1}^{\text {L}}=0$ and the high types bid $x_{1}^{\text {H}}=x_{2}^{\text {H}}=V^{\text {H}}/8$, which is just half of the expenditure in the corresponding contest with complete information. This trick may actually be generalized. Indeed, as can be checked, the proof of Theorem 1 continues to go through when we add “(iii)$p_{i,\omega }^{0}=1$” as a third alternative in Assumption (B). Then, it is easy to see that this relaxed variant of Assumption (B) can always be satisfied by modifying the relevant parameters, so that any player that is the only one having a positive budget in some state wins the contest in that state with certainty in the case of joint inactivity. In other words, Assumption (B) may be entirely dropped when one is willing to endogenize the sharing rule at the origin.

## Uniqueness

### Assumptions

As has been noted in prior work (e.g., Pérez-Castrillo and Verdier 1992; Cornes and Hartley 2005), standard concavity and convexity assumptions may be crucial for the uniqueness of PSNE in contests even under the assumption of complete information. Since we do not know of any general reason why asymmetric information should render uniqueness more likely in the present setup, we will keep Assumption (A). In addition, we will impose the following assumption on the information structure.

#### Assumption (C)

There is a mapping $v:\Omega \rightarrow {\mathbb {R}} _{++}\equiv (0,\infty )$ and, for each $i\in N$, a mapping $\kappa _{i} :\Theta _{i}\rightarrow {\mathbb {R}} _{++}$, such that $v_{i}(\omega )=v(\omega )\cdot \kappa _{i}(t_{i}(\omega ))$ for any $\omega \in \Omega$.

Assumption (C) requires that valuations can be expressed as a product of a common-value component (that may depend on the state, yet not on the player’s identity) and a private-value component (that may depend on a player’s identity and type, yet not directly on the state).^15^ The condition subsumes a large variety of commonly used information structures.

*(a) Independent private valuations* In this setting, the common-value component is trivial, i.e., $v(\omega )=1$ for any $\omega \in \Omega$, and valuations are stochastically independent across players. This setting clearly fulfills Assumption (C).

*(b) Correlated private values* In straightforward extension of the previous case, Assumption (C) is satisfied in settings in which each player knows her valuation at the time of bidding while valuations are *not* stochastically independent across players. Even the limit case of perfect correlation is covered, provided that zero-probability states are eliminated from the state space.

*(c) Pure common values* In still another setting, players share a common ex post valuation of the prize (e.g., because there is the possibility of resale), but players receive idiosyncratic signals. Thus, there is incomplete and potentially asymmetric information about the common valuation of the prize at the time of bidding. Assumption (C) holds because the private-value components may be set to one, i.e., $\kappa _{i}(\theta _{i})=1$ for any $i\in N$ and $\theta _{i}\in \Theta _{i}$.

*(d) Interdependent valuations* The case of interdependent valuations is consistent with Assumption (C), as Example 2A illustrates.

#### Example 2A

Assume that $v_{1}=\theta _{1}+\alpha \theta _{2}$ and $v_{2}=\theta _{2}+\alpha \theta _{1}$, with $\theta _{1}$ and $\theta _{2}$ independent, and $\alpha >0$ fixed. Provided that each player $i\in N=\{1,2\}$ has only two feasible type realizations $\theta ^{\text {H}}$ and $\theta ^{\text {L}}$ with $\theta ^{\text {H}}>\theta ^{\text {L}}>0$, Assumption (C) is satisfied. To see this, set the common-value component equal to

13 $$\begin{aligned} v(\omega )=\left\{ \begin{array}{ll} 1 &{} \text {if }t_{1}(\omega )\ne t_{2}(\omega )\\ \dfrac{(1+\alpha )\theta ^{\text {H}}}{\theta ^{\text {H}}+\alpha \theta ^{\text {L}}} &{} \text {if }t_{1}(\omega )=t_{2}(\omega )=\theta ^{\text {H}}\\ \dfrac{(1+\alpha )\theta ^{\text {L}}}{\theta ^{\text {L}}+\alpha \theta ^{\text {H}}} &{} \text {if }t_{1}(\omega )=t_{2}(\omega )=\theta ^{\text {L}}\text {,} \end{array} \right. \end{aligned}$$

and the private-value components equal to

14 $$\begin{aligned} \kappa _{i}(\theta _{i})=\left\{ \begin{array}{ll} \theta ^{\text {L}}+\alpha \theta ^{\text {H}} &{} \text {if }\theta _{i} =\theta ^{\text {L}}\\ \theta ^{\text {H}}+\alpha \theta ^{\text {L}} &{} \text {if }\theta _{i} =\theta ^{\text {H}}\text {.} \end{array} \right. \end{aligned}$$

Then, it can be checked in a straightforward way that $v_{i}(\omega )=v(\omega )\cdot \kappa _{i}(t_{i}(\omega ))$ for any $i\in N$ and $\omega \in \Omega$, as claimed.

However, as our next example shows, there are also settings that are not consistent with Assumption (C).

#### Example 2B

Consider a variation of the previous example in which each of the two players has three feasible type realizations $\theta ^{\text {H}}$, $\theta ^{\text {M}}$, and $\theta ^{\text {L}}$, with $\theta ^{\text {H}}>\theta ^{\text {M}}>\theta ^{\text {L}}>0$. Then, Assumption (C) implies $\kappa _{1}(\theta _{1})=\kappa _{2}(\theta _{2})$ if $\theta _{1}=\theta _{2}$.^16^ Moreover, dropping the superfluous index from $\kappa _{i}$,

15 $$\begin{aligned} \frac{\theta ^{\text {H}}+\alpha \theta ^{\text {L}}}{\theta ^{\text {L}} +\alpha \theta ^{\text {H}}}=\frac{\kappa (\theta ^{\text {H}})}{\kappa (\theta ^{\text {L}})}=\frac{\kappa (\theta ^{\text {H}})/\kappa (\theta ^{\text {M} })}{\kappa (\theta ^{\text {L}})/\kappa (\theta ^{\text {M}})}=\frac{(\theta ^{\text {H}}+\alpha \theta ^{\text {M}})(\theta ^{\text {M}}+\alpha \theta ^{\text {L} })}{(\theta ^{\text {M}}+\alpha \theta ^{\text {H}})(\theta ^{\text {L}}+\alpha \theta ^{\text {M}})}\text {,} \end{aligned}$$

which shows that Assumption (C) indeed fails to hold for any generic specification of the parameters. Similarly, with $n>2$ players, each of which has at least two feasible type realizations, and $v_{i}=\theta _{i}+\alpha {\textstyle \sum _{k\ne i}} \theta _{k}$, it is easy to see that Assumption (C) cannot be satisfied if $\alpha >0$ and $\alpha \ne 1$, because the ratio $v_{i}/v_{j}$, for $j\ne i$, will then depend on some $\theta _{k}$ with $k\ne i$ and $k\ne j$.

### The uniqueness result

The second main result of the present paper is the following.

#### Theorem 2

Under Assumptions (A) and (C), there exists at most one PSNE in the incomplete-information contest ${\mathcal {C}}$ . Moreover, there are no nondegenerate MSNE.

#### Proof

See the Appendix. $\square$

The proof of the first part of Theorem 2 is long and complicated. This is so because, as discussed in the Introduction, the methods introduced by Rosen (1965), Goodman (1980), and Ui (2008) need to be extended to deal with the case of discontinuous contests of the logit form. The following outline provides an overview of the proof.

**Fig. 2:**
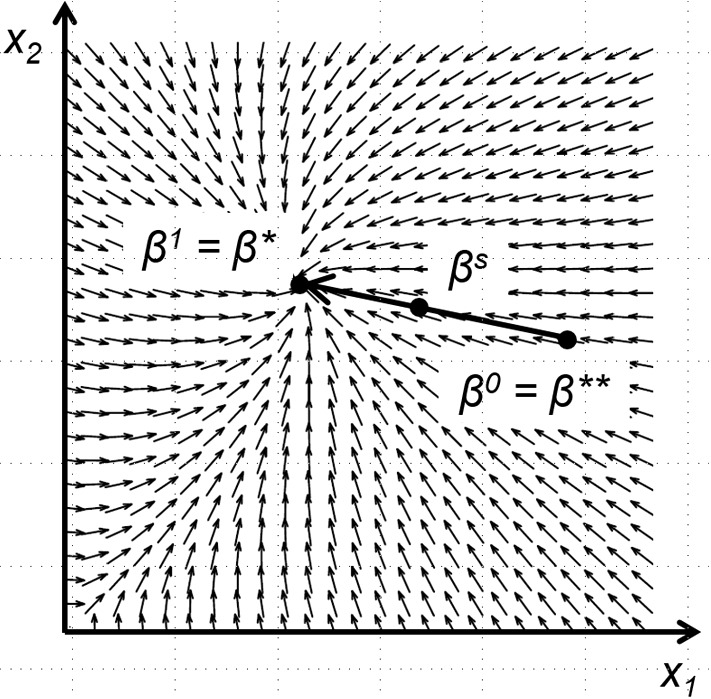
Illustration of the uniqueness proof

We start by noting that it suffices to prove the claim under the assumption of pure common values. Indeed, as can be checked, any incomplete-information contest satisfying Assumption (C) can be recast, via a simple rescaling of payoffs, as a contest with pure common values. Then, to provoke a contradiction, we assume the existence of two distinct equilibria $$\beta ^{*}$$ and $$\beta ^{**}$$, and consider the *inner product* of the vector $$\beta ^{*}-\beta ^{**}$$ with the payoff gradient in the agent normal form, where we replace any type’s marginal payoff by zero whenever her budget is zero. See also Fig. 2, which illustrates both the hypothetical vector $$\beta ^{*}-\beta ^{**}$$ and the concept of the payoff gradient.^17^ As a consequence of the Kuhn–Tucker conditions for payoff maximization, the inner product is zero or positive at $$\beta ^{*}$$, and zero or negative at $$\beta ^{**}$$.

To obtain a contradiction, we consider the straight path $$\beta ^{s}$$ that connects $$\beta ^{0}=\beta ^{**}$$ with $$\beta ^{1}=\beta ^{*}$$ (see again Fig. 2 for illustration) and claim that the derivative of the inner product at $$\beta ^{s}$$ with respect to *s* is negative. Now, the convexity of cost functions implies an upper bound for that derivative in terms of the Jacobian $$J_{p,\omega }$$ of the vector of marginal probabilities of winning at state $$\omega $$. Moreover, this upper bound is negative if the matrix sum $$J_{p,\omega }+J_{p,\omega }^{T}$$ is (i) negative semi-definite in all states of the world, and (ii) negative definite in at least one state $$\omega _{0}$$ in which $$\beta ^{*}$$ and $$\beta ^{**}$$ differ. To verify these conditions, we extend Goodman’s (1980) sufficient conditions by distinguishing between arbitrary nonzero bid profiles (for which $$J_{p,\omega }+J_{p,\omega }^{T}$$ can merely be shown to be negative semi-definite), and bid profiles that possess at least two nonzero components (for which $$J_{p,\omega }+J_{p,\omega }^{T}$$ can actually be shown to be negative definite). We then prove the existence of a state $$\omega _{0}$$ such that $$\beta ^{s}(t(\omega _{0}))$$ has at least two nonzero components for any $$s\in (0,1)$$, while $$\beta ^{*}$$ and $$\beta ^{**}$$ differ at $$\omega _{0}$$. Combining these observations, we can finally put a negative sign on the derivative of the inner product, and thereby obtain the desired contradiction.

The second part of Theorem 2 says that there cannot exist any Nash equilibrium in nondegenerate mixed strategies. This is more or less an immediate consequence of Assumption (A).^18^ Indeed, if type $\theta _{i}$ expects, with positive probability, a positive bid of at least one opponent, then her payoff function is strictly concave. If, however, type $\theta _{i}$ expects that all other players remain inactive with probability one, then her payoff function is strictly declining in the interior, which still implies strict quasiconcavity because $p_{i,\omega }\le 1$ for all $\omega \in {\mathcal {P}}_{i}(\theta _{i})$. It follows that any Nash equilibrium in the agent normal form must indeed be in pure strategies.^19^

## Symmetric contests

Additional results can be obtained for symmetric contests. For this, consider an incomplete-information contest ${\mathcal {C}}$ with state space $\Omega =\Theta _{1}\times \cdots \times \Theta _{n}$, where we assume $\Theta _{1}=\cdots =\Theta _{n}$, and where $t_{i}:\Omega \rightarrow \Theta _{i}$ is the projection on the *i*-th component, for any $i\in N$. Fix a *permutation* of the player set, i.e., a one-to-one mapping $\pi :N\rightarrow N$. Then, or any state $\omega =(\theta _{1},\ldots ,\theta _{n})\in \Omega$, let $\omega ^{\pi }=(\theta _{\pi (1)},\ldots ,\theta _{\pi (n)})$, so that in state $\omega ^{\pi }$, the type of player *i* is $t_{i}(\omega ^{\pi })=t_{\pi (i)}(\omega )=\theta _{\pi (i)}$. Similarly, for any profile of bids $x=(x_{1},\ldots ,x_{n})\in {\mathbb {R}} _{+}^{n}$, let$x^{\pi }=(x_{\pi (1)},\ldots ,x_{\pi (n)})$, so that in the profile $x^{\pi }$, player *i* chooses the bid $x_{\pi (i)}$. To define a symmetric contest, we will require that the primitives of the contest remain invariant under arbitrary permutations of the player set. Formally, an incomplete-information contest ${\mathcal {C}}$ as just introduced will be called *symmetric* if, for any $i\in N$, any $\omega \in \Omega$, and any permutation $\pi$, it holds that $q(\omega ^{\pi })=q(\omega )$, $f_{i,\omega ^{\pi }}(\cdot )=f_{\pi (i),\omega }(\cdot )$, $p_{i,\omega ^{\pi }}^{0} =p_{\pi (i),\omega }^{0}$, $v_{i}(\omega ^{\pi })=v_{\pi (i)}(\omega )$, $c_{i,\omega ^{\pi }}(\cdot )=c_{\pi (i),\omega }(\cdot )$, and $x_{i}^{\max } (\cdot )=x_{\pi (i)}^{\max }(\cdot )$ As detailed in the Appendix, these conditions jointly ensure that any symmetric contest of incomplete information is a symmetric *n*-player game.

Suppose that ${\mathcal {C}}$ is a symmetric incomplete-information contest. Let $\omega ^{\#}\in \Omega$ be a state and $i\in N$ be a player with positive budget in $\omega ^{\#}$, i.e., $x_{i}^{\max }(t_{i}(\omega ^{\#}))>0$. We will say that $\omega ^{\#}$ is *activity-inducing* for *i* if (ı) the contest is discontinuous in $\omega ^{\#}$, (ıı) there is at least one other player $j\in N\backslash \{i\}$ with a positive budget at $\omega ^{\#}$, and (ııı) any player $k\in N\backslash \{i\}$ with a positive budget at $\omega ^{\#}$ is of the same type as *i*.^20^ The following condition is a variant of Assumption (B) for symmetric contests.

### Assumption (B’)

For any $\omega \in \Omega$ and $i\in N$ such that $x_{i}^{\max }(t_{i}(\omega ))>0$, at least one of the following three conditions holds true:

(i) The contest is continuous in $\omega$;

(ii) There exists $j\in N\backslash \{i\}$ such that $x_{j}^{\max }(t_{j}(\omega ))>0$;

(iii) There exists a state $\omega ^{\#}\in {\mathcal {P}}_{i} (t_{i}(\omega ))$ that is activity-inducing for *i*.

Note that conditions (i) and (ii) are identical to those stated in Assumption (B). Hence, Assumption (B’) relaxes Assumption (B) in the sense that we may actually have a state $\omega \in \Omega$ in which both the contest is discontinuous and precisely one player $i\in N$ has a positive budget, provided that player *i* in state $\omega$ considers possible a (necessarily different) state $\omega ^{\#}\in \Omega$ that is activity-inducing for *i*.

Let ${\mathcal {C}}$ be a symmetric incomplete-information contest, as before. Then a profile of bid schedules $\beta =(\beta _{1},\ldots ,\beta _{n})\in B$ will be called *symmetric* if $\beta _{1}=\cdots =\beta _{n}$. The following theorem is the third and final main result of the present paper.

### Theorem 3

Consider a symmetric incomplete-information contest ${\mathcal {C}}$. Then, given Assumptions (A) and (B’), there exists a symmetric PSNE in ${\mathcal {C}}$. If, in addition, Assumption (C) holds, then the symmetric equilibrium is the unique PSNE.

### Proof

See the Appendix. $\square$

Thus, the assumptions for PSNE existence may indeed be relaxed in a symmetric contest. The idea of the proof should be clear by now. Should there be a state $\omega \in \Omega$ in which the contest is discontinuous and in which only one player $i\in N$ has a positive budget, then Assumption (B’) guarantees that this player deems possible a state $\omega ^{\#}\in \Omega$ at which all the endowed players (of which there are at least two) are in exactly the same strategic situation. Therefore, in state $\omega ^{\#}$, all the endowed players choose the same bid in any symmetric PSNE of the contest with minimum bid. Given that the contest is discontinuous in $\omega ^{\#}$, this common bid level (used also by player *i* at state $\omega$) remains bounded away from zero even when the minimum bid goes to zero. Hence, any limit profile of PSNE in the contests with minimum bid is a PSNE in the original contest.

It may be noted that it is really the symmetry of the contest that is crucial for the existence conclusion in Theorem 3, rather than the underlying information structure. Indeed, Example 1 assumes that $\Omega =\Theta _{1} \times \Theta _{2}$ with $\Theta _{1}=\Theta _{2}$, and that $t_{i}:\Omega \rightarrow \Theta _{i}$ is the projection on the *i*-th component, for any $i\in \{1,2\}$. Thus, the information structure assumed in Example 1 is as required by Theorem 3. However, the symmetry condition $x_{1}^{\max } (\theta ^{\text {L}})=x_{2}^{\max }(\theta ^{\text {L}})$ fails to hold, so that the contest is not symmetric. And indeed, as has been discussed, there may be no equilibrium in that contest.

The following example illustrates the usefulness of Theorem 3.

### Example 3

We modify Example 1 by assuming that the budgets of the low types are given by $x_{1}^{\max }(\theta ^{\text {L}})=x_{2}^{\max }(\theta ^{\text {L}})=0$, while the budgets of the high types remain as large as before. Then, Assumption (B) does not hold because in state $\omega _{\text {HL}}$, for instance, only player 1 has a positive budget. However, in contrast to Example 1, there is no problem with existence here because the contest is symmetric and Assumption (B’) holds. Indeed, $\omega _{\text {HH}} \in {\mathcal {P}}_{1}(\theta ^{\text {H}})\cap {\mathcal {P}}_{2}(\theta ^{\text {H}})$ is activity-inducing for both players, and hence, there is a symmetric PSNE (where $x_{1}^{\text {H}}=x_{2}^{\text {H}}=V^{\text {H}}/8$).

## Applications

This section reviews a number of specific settings in which Theorems 1 through 3 allow drawing new conclusions.

### Equilibria at the boundary

Hurley and Shogren (1998b) consider a two-player lottery contest with two types for one player and three types for the other player. They show that there is at most one interior PSNE. However, since their setting is one of private valuations (either independent or correlated), it follows from Theorems 1 and 2 that there is, in fact, exactly one PSNE. That equilibrium may be interior, but it may likewise be located at the boundary, as it happens, e.g., when low-valuation types submit zero bids in anticipation of aggressive bidding by the opponent. Thus, our results imply uniqueness regardless of boundary considerations.

### Asymmetric equilibria

An immediate consequence of the uniqueness result is that a symmetric contest satisfying Assumptions (A) and (C) does not admit any asymmetric equilibrium. Consider, for instance, the model of Malueg and Yates (2004), where each contestant $i\in N=\{1,2\}$ has the state-independent probability of winning

16 $$\begin{aligned} p_{i}(x_{1},x_{2})=\left\{ \begin{array}{ll} \dfrac{(x_{i})^{r}}{(x_{1})^{r}+(x_{2})^{r}} &{} \text {if }x_{1}+x_{2}>0\\ &{} \\ p_{i}^{0}\equiv \tfrac{1}{2} &{} \text {if }x_{1}+x_{2}=0\text {,} \end{array} \right. \end{aligned}$$

for some exogenous parameter $r>0$, while cost functions are linear, i.e., $c_{i}(x_{i})=x_{i}$. The state space is as in Example 1, but probabilities are now given by $q(\omega _{\text {LL}})=q(\omega _{\text {HH} })=\frac{\sigma }{2}$ and $q(\omega _{\text {LH}})=q(\omega _{\text {HL}} )=\frac{1-\sigma }{2}$, where $\sigma \in [0,1]$ is a correlation parameter. Malueg and Yates (2004) derive conditions necessary and sufficient for the existence of precisely one *symmetric* PSNE and show that these conditions hold, in particular, for $r\in (0,1]$. We may now refer to the discussion of the cases of independent and correlated private values following Theorem 2 to conclude that there is no asymmetric PSNE (nor MSNE) for $r=1$. There is likewise no asymmetric equilibrium for $r\in (0,1)$, since in this case, as discussed, a change of variables transforms the Tullock contest into a strategically equivalent lottery contest with state-independent cost functions ${\widetilde{c}}_{i}(y_{i})=(y_{i})^{1/r}$, to which our argument for $r=1$ extends in a straightforward way.

### Population uncertainty

In a contest with population uncertainty, the number of players entering the contest follows some exogenous probability distribution.^21^ Münster (2006), for example, characterizes the symmetric PSNE in a model with finitely many contestants each of which independently draws a zero or positive valuation. Strictly speaking, that setup does not satisfy our assumptions because there are states of the world in which not all players have a positive valuation. However, as discussed in Münster (2006, p. 355), it is inessential for the equilibrium analysis if inactivity is modeled as a lack of motivation (i.e., a zero valuation) or as a lack of endowment (i.e., a zero budget). Therefore, one may easily come up with an equivalent setup that satisfies the assumptions of Theorem 3. Thus, we have a unique PSNE. In fact, this conclusion remains true if stochastic participation is not necessarily independent across players.

### Contests with resale

Sui (2009) considers contests with the possibility of resale. In substance, this means that a winner with low valuation $V^{\text {L}}$ may offer the prize to the loser at price (marginally below) $V^{\text {H} }>V^{\text {L}}$, who will accept if and only if her valuation is $V^{\text {H} }$. Strictly speaking, Sui (2009) proves neither existence nor uniqueness.^22^ To apply Theorems 1 and 2, one notes that, in terms of continuation payoffs, winning is worth $V^{\text {L}}$ if both players have a low valuation, and $V^{\text {H}}$ otherwise. In other words, the possibility of resale creates a contest of pure common values. We conclude that there is indeed a unique PSNE in the reduced form of Sui’s game.

## Concluding remarks

As the preceding illustrations may have shown, the main results of the present paper provide a simple set of conditions sufficient for the existence of a unique PSNE in incomplete-information contests with or without budget constraints. While some of our assumptions could probably be further relaxed, the conditions are certainly general enough to deal with most applications that assume a discrete information structure.^23^

The analysis allows drawing potentially useful conclusions also regarding the expected net rent in rent-seeking games of incomplete information. To see why, note that for any *active* type $\theta _{i}$, i.e., for any type that chooses a positive bid, there is a state $\omega \in {\mathcal {P}}_{i}(\theta _{i})$ in which either the technology is continuous, or in which the technology is discontinuous but some player $j\ne i$ is active in $\omega$. Either way, $\theta _{i}$’s expected payoff is a strictly concave function of her own expenditure. Hence, noting that inactivity generates a (normalized) payoff of zero, equilibrium activity must yield a positive payoff. Thus, any player that is active with positive probability will realize a positive expected net rent.

An important problem for future work is comparative statics. Comparative statics for contests has been exhaustively studied in the case of complete information (see, e.g., Jensen 2016) and in the case of one-sided incomplete information (Hurley and Shogren 1998a). For two-sided asymmetric information, however, there are multiple effects that are not straightforward to disentangle (Hurley and Shogren 1998b). Consequently, it is not surprising that topics such as learning in contests (Pogrebna 2008; Aoyagi 2010), optimal bias (Drugov and Ryvkin 2017), approximate solutions (Gallice 2017), endogenous timing with asymmetric information (Fu 2006), transparency (Denter et al. 2011), the implications of changes to the information structure (Serena 2014; Denter and Sisak 2015), and information transmission (Slantchev 2010; Kovenock et al. 2015; Zhang and Zhou 2016) have recently become very active fields of research.

There are several dimensions in which the present analysis could be usefully extended. For instance, we did not consider productive effort (Chung 1996; Chowdhury and Sheremeta 2011; Hirai and Szidarovszky 2013), nor group contests (Fu et al. 2015; Brookins and Ryvkin 2016).^24^ Some generalization would be desirable also for the existence part. So far, the direct application of Reny’s theorem to incomplete-information contests has primarily been an expositional alternative. However, new results of practical relevance for contest theory might be feasible by combining arguments specific to contests with the insights of the more recent literature on equilibrium existence in discontinuous games (e.g., Carbonell-Nicolau and McLean 2017; He and Yannelis 2015, 2016).

## Appendix. Proofs

This appendix contains the proofs of Theorems 1, 2, and 3. The proofs are based on altogether eight lemmas. Lemma A.1 is used in the proof of Theorems 1 and 3, while Lemma A.2 is used in the proof of Theorem 2. Lemmas A.3 through A.7 enter the proof of Lemma A.2 (where the use of Lemma A.5 is indirect via Lemma A.4). Lemma A.8, finally, is employed in the proof of Theorem 3.

### Proof of Theorem 1

Let ${\overline{\varepsilon }}>0$ be small enough such that ${\overline{\varepsilon }}<x_{i}^{\max }(\theta _{i})$ for any$\ i\in N$ and $\theta _{i}\in \Theta _{i}$ with $x_{i}^{\max }(\theta _{i})>0$. Then, for any $\varepsilon \in (0,{\overline{\varepsilon }})$, restricting the choice of any type $\theta _{i}\in \Theta _{i}$ with $x_{i}^{\max }(\theta _{i})>0$ to $[\varepsilon ,x_{i}^{\max }(\theta _{i})]$, for any $i\in N$, defines the *contest with minimum bid*, ${\mathcal {C}}(\varepsilon )$. By Assumption (A), type $\theta _{i}$’s expected payoff in ${\mathcal {C}}(\varepsilon )$ is concave in her own bid, as well as continuous in the profile of bid schedules. Hence, by the Nikaidô and Isoda (1955) theorem, a PSNE exists in the agent normal form of ${\mathcal {C}}(\varepsilon )$. Fix now a sequence $\{\varepsilon _{m}\}_{m=1}^{\infty }$ in $(0,{\overline{\varepsilon }})$ with $\lim _{m\rightarrow \infty }\varepsilon _{m}=0$, and select a PSNE $\beta ^{m}$ in ${\mathcal {C}}(\varepsilon _{m})$, for each $m\in \{1,2,\ldots \}$. Since *B*, as defined in Sect. 3, is a compact subset of Euclidean space, we may assume w.l.o.g. that the sequence $\{\beta ^{m}\}_{m=1}^{\infty }$ converges to some profile of bid schedules $\beta ^{*}\in B$. We claim that $\beta ^{*}$ is a PSNE in the original contest ${\mathcal {C}}$. For this, take any $i\in N$ and $\theta _{i}\in \Theta _{i}$ such that $x_{i}^{\max }(\theta _{i})>0$, and consider a deviation $x_{i}\in [0,x_{i}^{\max }(\theta _{i})]$.

*Case 1.* Suppose first that $x_{i}>0$. Then, for any *m* sufficiently large, $x_{i}\ge \varepsilon _{m}$, so that, by the optimality of type $\theta _{i}$’s bid $\beta _{i}^{m}(\theta _{i})$ in ${\mathcal {C}} (\varepsilon _{m})$,

17 $$\begin{aligned}&{\sum \limits _{\omega \in {\mathcal {P}}_{i}(\theta _{i})}} q_{i}(\omega \vert \theta _{i})\Pi _{i,\omega }(\beta _{i}^{m}(\theta _{i}),\beta _{-i}^{m} (t_{-i}(\omega ))) \nonumber \\&\quad \ge {\sum \limits _{\omega \in {\mathcal {P}}_{i}(\theta _{i})}} q_{i}(\omega \vert \theta _{i})\Pi _{i,\omega }(x_{i},\beta _{-i}^{m}(t_{-i}(\omega )))\text {.} \end{aligned}$$

Fix any $\omega \in {\mathcal {P}}_{i}(\theta _{i})$. By Assumption (B), either (i) the contest is continuous in $\omega$, or (ii) there exists $j\in N\backslash \{i\}$ such that $x_{j}^{\max }(t_{j}(\omega ))>0$. In case (i), the function $\Pi _{i,\omega }(\cdot )$ is continuous at $\beta ^{*}(t(\omega ))$ by Assumption (A). In case (ii), we may assume w.l.o.g. that the contest is discontinuous in $\omega$. But then, Lemma A.1 implies that $\beta ^{*}(t(\omega ))\ne 0$, so that $\Pi _{i,\omega }(\cdot )$ is likewise continuous at $\beta ^{*}(t(\omega ))$. Recalling that $x_{i}>0$, and letting $m\rightarrow \infty$ in (17) yields ${\overline{\Pi }}_{i}(\beta _{i} ^{*}(\theta _{i}),\beta _{-i}^{*},\theta _{i})\ge {\overline{\Pi }}_{i} (x_{i},\beta _{-i}^{*},\theta _{i})$, i.e., $x_{i}$ is not a profitable deviation for $\theta _{i}$.

*Case 2.* Suppose next that $x_{i}=0$. Clearly, for any small but positive $\delta >0$,

18 $$\begin{aligned} {\overline{\Pi }}_{i}(\beta _{i}^{*}(\theta _{i}),\beta _{-i}^{*},\theta _{i})\ge {\overline{\Pi }}_{i}(\delta ,\beta _{-i}^{*},\theta _{i})\text {,} \end{aligned}$$

as just seen. But since $p_{i,\omega }(\cdot ,x_{-i})$ is nondecreasing for any $\omega \in \Omega$ and $x_{-i}\in {\mathbb {R}} _{+}^{n-1}$, we obtain the lower bound

19 $$\begin{aligned} {\overline{\Pi }}_{i}(\delta ,\beta _{-i}^{*},\theta _{i})\ge {\overline{\Pi }} _{i}(0,\beta _{-i}^{*},\theta _{i})-E\left[ c_{i,\omega }(\delta )\right] +E\left[ c_{i,\omega }(0)\right] \text {,} \end{aligned}$$

where expectations are taken with respect to $q_{i}(\cdot \vert \theta _{i})$. Combining inequalities (18) and (19), and subsequently letting $\delta \rightarrow 0$, the continuity of cost functions implies that ${\overline{\Pi }}_{i}(\beta _{i}^{*}(\theta _{i}),\beta _{-i} ^{*},\theta _{i})\ge {\overline{\Pi }}_{i}(0,\beta _{-i}^{*},\theta _{i})$. Thus, a deviation to $x_{i}=0$ is not profitable either.

In sum, type $\theta _{i}$ has no profitable deviation. Since $i\in N$ and $\theta _{i}\in \Theta _{i}$ with $x_{i}^{\max }(\theta _{i})>0$ were arbitrary, $\beta ^{*}$ is indeed a PSNE in ${\mathcal {C}}$. $\square$

The following lemma reflects the key intuition underlying the existence argument. Since the lemma will be used also in the proof of Theorem 3, we remark that its proof does not make use of Assumption (B).

### Lemma A.1

Let $\omega \in \Omega$ and players $i,j\in N$ with $i\ne j$ such that $x_{i}^{\max } (t_{i}(\omega ))>0$ and $x_{j}^{\max }(t_{j}(\omega ))>0$. If the contest is discontinuous in $\omega$, then $\beta ^{*}(t(\omega ))\ne 0$.

### Proof

Clearly, $p_{i,\omega }(\beta ^{m}(t(\omega )))+p_{j,\omega }(\beta ^{m}(t(\omega )))\le 1$ for any *m*. Hence, possibly after exchanging *i* and *j*, we have that $p_{i,\omega }(\beta ^{m} (t(\omega )))\le \frac{1}{2}$ holds for infinitely many *m*. By transition to a suitable subsequence, we may assume w.l.o.g. that this is true for any *m*. But then,

20 $$\begin{aligned} \frac{\partial p_{i,\omega }(\beta ^{m}(t(\omega )))}{\partial x_{i}}&=\frac{\partial f_{i,\omega }(\beta _{i}^{m}(t_{i}(\omega )))/\partial x_{i} }{f_{1,\omega }(\beta _{1}^{m}(t_{1}(\omega )))+\cdots +f_{n,\omega }(\beta _{n} ^{m}(t_{n}(\omega )))}\cdot (1-p_{i,\omega }(\beta ^{m}(t(\omega )))) \end{aligned}$$

21 $$\begin{aligned}&\ge \frac{\partial f_{i,\omega }(\beta _{i}^{m}(t_{i}(\omega )))/\partial x_{i}}{f_{1,\omega }(\beta _{1}^{m}(t_{1}(\omega )))+\cdots +f_{n,\omega }(\beta _{n}^{m}(t_{n}(\omega )))}\cdot \frac{1}{2}\text { ,} \end{aligned}$$

for any *m*. To provoke a contradiction, suppose now that $\lim _{m\rightarrow \infty }\beta ^{m}(t(\omega ))=0$. Then, taking into account that $\partial f_{i,\omega }(0)/\partial x_{i}>0$, the RHS of inequality (21) tends to infinity as $m\rightarrow \infty$. Therefore, for type $\theta _{i}=t_{i} (\omega )$, positive valuations and bounded marginal costs imply that $\partial {\overline{\Pi }}_{i}(\beta _{i}^{m}(\theta _{i}),\beta _{-i}^{m} ,\theta _{i})/\partial x_{i}>0$ holds for any sufficiently large *m*. For any such *m*, the necessary Kuhn–Tucker conditions imply $\beta _{i}^{m}(\theta _{i})=x_{i}^{\max }(\theta _{i})$, in conflict with $\lim _{m\rightarrow \infty }\beta _{i}^{m}(\theta _{i})=0$. The lemma follows. $\square$

### Proof of Theorem 2

We show first that there is at most one PSNE. Let $i\in N$ and $\theta _{i}\in \Theta _{i}$. Then, by Assumption (C), maximizing $\theta _{i}$’s expected payoff

22 $$\begin{aligned} {\overline{\Pi }}_{i}(x_{i},\beta _{-i},\theta _{i})= {\sum _{\omega \in {\mathcal {P}}_{i}(\theta _{i})}} q_{i}(\omega \vert \theta _{i})\left\{ p_{i,\omega }(x_{i},\beta _{-i}(t_{-i}(\omega )))v_{i} (\omega )-c_{i,\omega }(x_{i})\right\} \end{aligned}$$

is equivalent to maximizing $\theta _{i}$’s expected payoff in units of her private-valuation component, $\kappa _{i}(\theta _{i})>0$. Formally,

23 $$\begin{aligned} \frac{{\overline{\Pi }}_{i}(x_{i},\beta _{-i},\theta _{i})}{\kappa _{i}(\theta _{i} )}{=} {\sum _{\omega \in {\mathcal {P}}_{i}(\theta _{i})}} q_{i}(\omega \vert \theta _{i})\left( p_{i,\omega }(x_{i},\beta _{-i}(t_{-i}(\omega )))v(\omega )-\frac{c_{i,\omega }(x_{i})}{\kappa _{i}(t_{i}(\omega ))}\right) \text {.} \quad \quad \end{aligned}$$

Thus, a *normalized contest*$\widehat{{\mathcal {C}}}$, strategically equivalent to ${\mathcal {C}}$, may be characterized by valuations $\widehat{v}_{i}(\omega )=v(\omega )$ and cost functions ${\widehat{c}}_{i,\omega } (x_{i})=c_{i,\omega }(x_{i})/\kappa _{i}(t_{i}(\omega ))$. Clearly, $\widehat{{\mathcal {C}}}$ is a contest with a pure common valuation that satisfies Assumption (A). Therefore, by Lemma A.2, there is at most one PSNE in $\widehat{{\mathcal {C}}}$. It follows that there is indeed at most one PSNE in ${\mathcal {C}}$. To prove the second claim, let ${\mathfrak {b}}^{*}=({\mathfrak {b}}_{1}^{*},\ldots ,{\mathfrak {b}}_{n}^{*})$ be a MSNE in ${\mathcal {C}}$. Fix $i\in N$ and $\theta _{i}\in \Theta _{i}$. Then $\theta _{i}$’s expected payoff from a bid $x_{i}\ge 0$ is given by

24 $$\begin{aligned} {\overline{\Pi }}_{i}(x_{i},{\mathfrak {b}}_{-i}^{*},\theta _{i})= {\sum _{\omega \in {\mathcal {P}}_{i}(\theta _{i})}} q_{i}(\omega \vert \theta _{i})E_{{\mathfrak {b}}_{-i}^{*}(t_{-i}(\omega ))}\left[ \Pi _{i,\omega }(x_{i},x_{-i})\right] \text {,} \end{aligned}$$

where the expectation in state $\omega$ is taken over the realizations of $x_{-i}$ according to the product measure ${\mathfrak {b}}_{-i}^{*} (t_{-i}(\omega ))=\{{\mathfrak {b}}_{j}^{*}(t_{j}(\omega ))\}_{j\ne i}$. By Assumption (A), $\Pi _{i,\omega }(\cdot ,x_{-i})$ is concave for any $x_{-i}\in {\mathbb {R}} _{+}^{n-1}$, and even strictly so if $x_{-i}\ne 0$. Therefore, if for some $\omega \in {\mathcal {P}}_{i}(\theta _{i})$ and $j\in N\backslash \{i\}$, the support of ${\mathfrak {b}}_{j}^{*}(t_{j}(\omega ))$ contains a positive bid, then the mapping $x_{i}\mapsto {\overline{\Pi }}_{i}(x_{i},{\mathfrak {b}}_{-i} ^{*},\theta _{i})$ is strictly concave on ${\mathbb {R}} _{+}$. Hence, the probability distribution ${\mathfrak {b}}_{i}^{*}(\theta _{i})$ must be degenerate. If, however, for any $\omega \in {\mathcal {P}} _{i}(\theta _{i})$ and $j\in N\backslash \{i\}$, the probability measure ${\mathfrak {b}}_{j}^{*}(t_{j}(\omega ))$ assigns all probability mass to the zero bid, then $x_{i}\mapsto {\overline{\Pi }}_{i}(x_{i},{\mathfrak {b}}_{-i}^{*},\theta _{i})$ is, after a potential upward jump at the zero bid, strictly declining in the interior by Assumption (A). Thus, also in this case, ${\mathfrak {b}}_{i}^{*}(\theta _{i})$ must be degenerate. Since $i\in N$ and $\theta _{i}\in \Theta _{i}$ were arbitrary, this proves the second claim. The theorem follows. $\square$

The lemma below deals with the case of a pure common valuation.

### Lemma A.2

If $\kappa _{i}(\theta _{i})=1$ for all $i\in N$ and $\theta _{i}\in \Theta _{i}$, then there is at most one PSNE.

### Proof

Suppose there are PSNE $$\beta ^{*}$$ and $$\beta ^{**}$$, with $$\beta ^{*}\ne \beta ^{**}$$. Consider the straight path $$\beta ^{s}=s\beta ^{*}+(1-s)\beta ^{**}$$, where $$s\in [0,1]$$. Then, as illustrated in Fig. 2, $$\beta ^{0}=\beta ^{**}$$ and $$\beta ^{1}=\beta ^{*}$$. Let $$i\in N$$ and $$\theta _{i} \in \Theta _{i}$$. Then, by Lemma A.3, we may define type $$\theta _{i}$$’s “marginal interim payoff” at the strategy profile $$\beta ^{s}$$ as

25 $$\begin{aligned} {\overline{\pi }}_{i}(s,\theta _{i})=\left\{ \begin{array}{ll} \dfrac{\partial {\overline{\Pi }}_{i}(\beta _{i}^{s}(\theta _{i}),\beta _{-i} ^{s},\theta _{i})}{\partial x_{i}} &{} \text {if }x_{i}^{\max }(\theta _{i})>0\\ &{} \\ 0 &{} \text {if }x_{i}^{\max }(\theta _{i})=0\text {.} \end{array} \right. \end{aligned}$$

Consider now the inner product

26 $$\begin{aligned} \gamma _{s}=\sum _{i=1}^{n}\sum _{\theta _{i}\in \Theta _{i}}q_{i}(\theta _{i})\{\beta _{i}^{*}(\theta _{i})-\beta _{i}^{**}(\theta _{i} )\}{\overline{\pi }}_{i}(s,\theta _{i})\text {.} \end{aligned}$$

Then, at $$s=0$$, the necessary Kuhn–Tucker conditions for type $$\theta _{i}$$ at the equilibrium $$\beta ^{0}=\beta ^{**}$$ imply $$\beta _{i}^{**}(\theta _{i})=0$$ if $${\overline{\pi }}_{i}(0,\theta _{i})<0$$, and $$\beta _{i} ^{**}(\theta _{i})=x_{i}^{\max }(\theta _{i})$$ if $${\overline{\pi }} _{i}(0,\theta _{i})>0$$. It follows that $$\gamma _{0}\le 0$$. Similarly, the necessary Kuhn–Tucker conditions at the equilibrium $$\beta ^{1}=\beta ^{*}$$ imply that $$\gamma _{1}\ge 0$$. To provoke a contradiction, we will now show that $$\gamma _{s}$$ is strictly declining over the interval [0, 1]. Combining Eq. (25) with (37) from Lemma A.3 delivers

27 $$\begin{aligned} {\overline{\pi }}_{i}(s,\theta _{i})= {\sum _{\omega \in {\mathcal {P}}_{i}(\theta _{i})}} q_{i}(\omega \vert \theta _{i})\pi _{i,\omega }(s)\text {,} \end{aligned}$$

where player *i*’s “marginal ex post payoff” at state $\omega$ is given by

28 $$\begin{aligned} \pi _{i,\omega }(s)=\left\{ \begin{array} [c]{ll} \dfrac{\partial \Pi _{i,\omega }(\beta ^{s}(t(\omega )))}{\partial x_{i}} &{} \text {if }x_{i}^{\max }(t_{i}(\omega ))>0\\ &{} \\ 0 &{} \text {if }x_{i}^{\max }(t_{i}(\omega ))=0\text {.} \end{array} \right. \end{aligned}$$

Plugging (27) into (26), and exploiting that $\omega \in {\mathcal {P}}_{i}(\theta _{i})$ implies $q_{i}(\theta _{i})q_{i}(\omega \vert \theta _{i})=q(\omega )$, we find that

29 $$\begin{aligned} \gamma _{s}=\sum _{i=1}^{n}\sum _{\theta _{i}\in \Theta _{i}}\sum _{\omega \in {\mathcal {P}}_{i}(\theta _{i})}q(\omega )\{\beta _{i}^{*}(\theta _{i} )-\beta _{i}^{**}(\theta _{i})\}\pi _{i,\omega }(s)\text {.} \end{aligned}$$

Since player *i*’s possibility sets ${\mathcal {P}}_{i}(\theta _{i})$, for signals $\theta _{i}$ ranging over the type space $\Theta _{i}$, form a partition of the state space $\Omega$, this may be written more compactly as

30 $$\begin{aligned} \gamma _{s}=\sum _{i=1}^{n}\sum _{\omega \in \Omega }q(\omega )z_{i}(\omega )\pi _{i,\omega }(s)\text {,} \end{aligned}$$

where $$z_{i}(\omega )=\beta _{i}^{*}(t_{i}(\omega ))-\beta _{i}^{**}(t_{i}(\omega ))$$. Fix $$i\in N$$ and $$\omega \in \Omega $$ for the moment. We claim that $$\pi _{i,\omega }(\cdot )$$ is differentiable. It should be clear that, to prove the claim, we may assume w.l.o.g. that (i) $$x_{i}^{\max }(t_{i} (\omega ))>0$$, (ii) the contest is discontinuous in $$\omega $$, and (iii) $$\beta ^{*}(t(\omega ))=0$$ or $$\beta ^{**}(t(\omega ))=0$$. But under these conditions, since $$p_{1,\omega }^{0}+\cdots +p_{n,\omega }^{0}\le 1$$, player *i* is the only player with a positive budget at $$\omega $$. Moreover, $$p_{i,\omega }^{0}=1$$. Therefore, $$\Pi _{i,\omega }(x_{i},\beta _{-i}^{s} (t(\omega )))=v_{i}(\omega )-c_{i,\omega }(x_{i})$$, so that $$\pi _{i,\omega }(s)=-\partial c_{i,\omega }(\beta _{i}^{s}(t(\omega )))/\partial x_{i}$$ is indeed differentiable. It follows that (30) is differentiable as well, with

31 $$\begin{aligned} \frac{\partial \gamma _{s}}{\partial s}=\sum _{i=1}^{n}\sum _{\omega \in \Omega }q(\omega )z_{i}(\omega )\frac{\partial \pi _{i,\omega }(s)}{\partial s}\text {.} \end{aligned}$$

To determine the sign of (31), fix some $i\in N$ and $\omega \in \Omega$. If $x_{i}^{\max }(t_{i}(\omega ))>0$, then

32 $$\begin{aligned} \pi _{i,\omega }(s)=v_{i}(\omega )\frac{\partial p_{i,\omega }(\beta _{1}^{s} (t_{1}(\omega )),\ldots ,\beta _{n}^{s}(t_{n}(\omega )))}{\partial x_{i}} -\frac{\partial c_{i,\omega }(\beta _{i}^{s}(t_{i}(\omega )))}{\partial x_{i} }\text {,} \end{aligned}$$

and an application of the chain rule for differentiation, using $$\beta _{j} ^{s}(t_{j}(\omega ))=\beta _{j}^{**}(t_{j}(\omega ))+s\cdot z_{j}(\omega )$$ for $$j\in N$$, leads to

33 $$\begin{aligned} \frac{\partial \pi _{i,\omega }(s)}{\partial s}&=v_{i}(\omega )\sum _{j=1} ^{n}\frac{\partial ^{2}p_{i,\omega }(\beta _{1}^{s}(t_{1}(\omega )),\ldots ,\beta _{n}^{s}(t_{n}(\omega )))}{\partial x_{j}\partial x_{i}}z_{j}(\omega )\nonumber \\&\quad -\frac{\partial ^{2}c_{i,\omega }(\beta _{i}^{s}(t_{i}(\omega )))}{\partial x_{i}^{2}}z_{i}(\omega )\text {.} \end{aligned}$$

Multiplying Eq. (33) through with $z_{i}(\omega )$, and subsequently exploiting the convexity of cost functions, one obtains

34 $$\begin{aligned} z_{i}(\omega )\frac{\partial \pi _{i,\omega }(s)}{\partial s}\le v_{i} (\omega )\sum _{j=1}^{n}\frac{\partial ^{2}p_{i,\omega }(\beta ^{s}(t(\omega )))}{\partial x_{j}\partial x_{i}}z_{i}(\omega )z_{j}(\omega )\text {.} \end{aligned}$$

If, however, $$x_{i}^{\max }(t_{i}(\omega ))=0$$, then $$z_{i}(\omega )=\beta _{i}^{**}(t_{i}(\omega ))-\beta _{i}^{*}(t_{i}(\omega ))=0$$, and (34) is likewise satisfied. Thus, (34) holds for any $$i\in N$$ and $$\omega \in \Omega $$. Combining the inequality with (31), and exploiting that $$v_{i}(\cdot )=v(\cdot )$$ for any $$i\in N$$, one arrives at

35 $$\begin{aligned} \frac{\partial \gamma _{s}}{\partial s}&\le \sum _{i=1}^{n}\sum _{\omega \in \Omega }q(\omega )v_{i}(\omega )\sum _{j=1}^{n}\frac{\partial ^{2}p_{i,\omega }(\beta ^{s}(t(\omega )))}{\partial x_{j}\partial x_{i}}z_{i}(\omega )z_{j}(\omega ) \end{aligned}$$

36 $$\begin{aligned}&=\sum _{\omega \in \Omega }q(\omega )v(\omega )z(\omega )^{T}J_{p,\omega } (\beta ^{s}(t(\omega )))z(\omega )\text {,} \end{aligned}$$

where $$z(\omega )=(z_{1}(\omega ),\ldots ,z_{n}(\omega ))^{T}$$, and $$J_{p,\omega } (x)$$ is the $$n\times n$$-matrix whose element at the intersection of row $$i\in N$$ and column $$j\in N$$ is $$\partial ^{2}p_{i,\omega }(x)/\partial x_{j}\partial x_{i}$$. Since we know that $$\gamma _{s}$$ is (differentiable, hence) continuous on [0, 1], it suffices to show that the right-hand side of (36) is negative for any $$s\in (0,1)$$. So assume that $$s\in (0,1)$$. Let $$\Omega _{0}=\{\omega \in \Omega :z(\omega )\ne 0\}$$ denote the set of states at which $$\beta ^{*}$$ and $$\beta ^{**}$$ differ. Then, for any $$\omega \in \Omega _{0}$$, we clearly have $$\beta ^{s}(t(\omega ))\ne 0$$. Hence, by Lemma A.4, $$J_{p,\omega }(\beta ^{s}(t(\omega )))+J_{p,\omega }(\beta ^{s}(t(\omega )))^{T}$$ is negative semi-definite for any $$\omega \in \Omega _{0}$$. Thus, using Lemma A.6, $$z(\omega )^{T}J_{p,\omega }(\beta ^{s}(t(\omega )))z(\omega )\le 0$$, for any $$\omega \in \Omega _{0}$$. Moreover, by Lemma A.7, there is a state $$\omega _{0}\in \Omega _{0}$$ such that the bid profile $$\beta ^{s}(t(\omega _{0}))\in {\mathbb {R}} _{+}^{n}$$ has at least two nonzero components. Thus, using the strict versions of Lemmas A.4 and A.6, the right-hand side of (36) is indeed seen to be negative. $$\square $$

The lemma below is concerned with the differentiability of interim expected payoffs.

### Lemma A.3

If $x_{i}^{\max }(\theta _{i})>0$, then the function $x_{i}\mapsto {\overline{\Pi }}_{i}(x_{i},\beta _{-i}^{s} ,\theta _{i})$ is differentiable at $x_{i}=\beta _{i}^{s}(\theta _{i} )$ for any $s\in [0,1]$, with

37 $$\begin{aligned} \frac{\partial {\overline{\Pi }}_{i}(\beta _{i}^{s}(\theta _{i}),\beta _{-i} ^{s},\theta _{i})}{\partial x_{i}}= {\sum _{\omega \in {\mathcal {P}}_{i}(\theta _{i})}} q_{i}(\omega \vert \theta _{i})\frac{\partial \Pi _{i,\omega }(\beta _{i}^{s}(\theta _{i}),\beta _{-i}^{s}(t_{-i}(\omega )))}{\partial x_{i}}\text {.} \end{aligned}$$

### Proof

Take some $$s\in [0,1]$$. In view of Eq. (3), it suffices to show that $$\Pi _{i,\omega }(x_{i},\beta _{-i} ^{s}(t_{-i}(\omega )))$$ is differentiable at$$x_{i}=\beta _{i} ^{s}(\theta _{i})$$, for any $$\omega \in {\mathcal {P}}_{i}(\theta _{i})$$. So let $$\omega \in {\mathcal {P}}_{i}(\theta _{i})$$. To provoke a contradiction, suppose that $$\partial \Pi _{i,\omega }(\beta ^{s}(t(\omega )))/\partial x_{i}$$ does not exist. Then, necessarily, $$\beta ^{s}(t(\omega ))=0$$. Moreover, $$f_{1,\omega }(0)+\cdots +f_{n,\omega }(0)=0$$, and $$p_{i,\omega }^{0}<1$$. Assume first that $$s=1$$, so that $$\beta ^{s}=\beta ^{*}$$ and, hence, $$\beta ^{*} (t(\omega ))=0$$. Then, since $$x_{i}^{\max }(\theta _{i})>0$$, type $$\theta _{i}$$’s inactivity cannot be optimal by the usual discontinuity argument. Assume next that $$s<1$$. Then $$\beta ^{s}(t(\omega ))=0$$ implies $$\beta ^{**} (t(\omega ))=0$$, which is likewise impossible. This proves the lemma. $$\square $$

The next lemma extends Goodman’s (1980) argument.

### Lemma A.4

For any $\omega \in \Omega$, the matrix $J_{p,\omega }(x)+J_{p,\omega }(x)^{T}$ is negative semi-definite for any bid profile $x\in {\mathbb {R}} _{+}^{n}\backslash \{0\}$, and negative definite for any bid profile $x\in {\mathbb {R}} _{+}^{n}$ possessing at least two nonzero components.

### Proof

Take any $\omega \in \Omega$ and $x\in {\mathbb {R}} _{+}^{n}\backslash \{0\}$. We wish to show that the matrix sum

38 $$\begin{aligned}&J_{p,\omega }(x)+J_{p,\omega }(x)^{T}\nonumber \\&=\left( \begin{array} [c]{cccc} 2\frac{\partial ^{2}p_{1,\omega }(x)}{\partial x_{1}^{2}} &{} \frac{\partial ^{2}p_{1,\omega }(x)}{\partial x_{2}\partial x_{1}}+\frac{\partial ^{2}p_{2,\omega }(x)}{\partial x_{1}\partial x_{2}} &{} \cdots &{} \frac{\partial ^{2}p_{1,\omega }(x)}{\partial x_{n}\partial x_{1}}+\frac{\partial ^{2}p_{n,\omega }(x)}{\partial x_{1}\partial x_{n}}\\ \frac{\partial ^{2}p_{2,\omega }(x)}{\partial x_{1}\partial x_{2}} +\frac{\partial ^{2}p_{1,\omega }(x)}{\partial x_{2}\partial x_{1}} &{} 2\frac{\partial ^{2}p_{2,\omega }(x)}{\partial x_{2}^{2}} &{} \cdots &{} \frac{\partial ^{2}p_{2,\omega }(x)}{\partial x_{n}\partial x_{2}} +\frac{\partial ^{2}p_{n,\omega }(x)}{\partial x_{2}\partial x_{n}}\\ \vdots &{} \vdots &{} \ddots &{} \vdots \\ \frac{\partial ^{2}p_{n,\omega }(x)}{\partial x_{1}\partial x_{n}} +\frac{\partial ^{2}p_{1,\omega }(x)}{\partial x_{n}\partial x_{1}} &{} \frac{\partial ^{2}p_{n,\omega }(x)}{\partial x_{2}\partial x_{n}} +\frac{\partial ^{2}p_{2,\omega }(x)}{\partial x_{n}\partial x_{2}} &{} \cdots &{} 2\frac{\partial ^{2}p_{n,\omega }(x)}{\partial x_{n}^{2}} \end{array} \right) \end{aligned}$$

is negative semi-definite. By Assumption (A), $\partial ^{2}p_{i,\omega }(x)/\partial x_{i}^{2}\le 0$, for any $i\in N$. Therefore, the diagonal matrix

39 $$\begin{aligned} M_{\omega }(x)=\left( \begin{array} [c]{cccc} \frac{\partial ^{2}p_{1,\omega }(x)}{\partial x_{1}^{2}} &{} 0 &{} \cdots &{} 0\\ 0 &{} \frac{\partial ^{2}p_{2,\omega }(x)}{\partial x_{2}^{2}} &{} &{} \vdots \\ \vdots &{} &{} \ddots &{} 0\\ 0 &{} \cdots &{} 0 &{} \frac{\partial ^{2}p_{n,\omega }(x)}{\partial x_{n}^{2}} \end{array} \right) \end{aligned}$$

is negative semi-definite. Consider now some $k\in N$. If $f_{k,\omega } (x_{k})>0$, then Lemma A.5 implies that the mapping $${\widetilde{x}} _{-k}\mapsto p_{k,\omega }(x_{k},{\widetilde{x}}_{-k})\ $$is convex on ${\mathbb {R}} _{+}^{n-1}$. Moreover, if $f_{k,\omega }(x_{k})=0$, then the mapping $${\widetilde{x}}_{-k}\mapsto p_{k,\omega }(x_{k},{\widetilde{x}}_{-k})\ $$equals zero on ${\mathbb {R}} _{+}^{n-1}\backslash \{0\}$. Either way, the corresponding Hessian,

40 $$\begin{aligned} H_{k,\omega }(x)=\left( \begin{array} [c]{cccccc} \frac{\partial ^{2}p_{k,\omega }(x)}{\partial x_{1}^{2}} &{} \cdots &{} \frac{\partial ^{2}p_{k,\omega }(x)}{\partial x_{k-1}\partial x_{1}} &{} \frac{\partial ^{2}p_{k,\omega }(x)}{\partial x_{k+1}\partial x_{1}} &{} \cdots &{} \frac{\partial ^{2}p_{k,\omega }(x)}{\partial x_{n}\partial x_{1}}\\ \vdots &{} \ddots &{} \vdots &{} \vdots &{} &{} \vdots \\ \frac{\partial ^{2}p_{k,\omega }(x)}{\partial x_{1}\partial x_{k-1}} &{} \cdots &{} \frac{\partial ^{2}p_{k,\omega }(x)}{\partial x_{k-1}^{2}} &{} \frac{\partial ^{2}p_{k,\omega }(x)}{\partial x_{k+1}\partial x_{k-1}} &{} \cdots &{} \frac{\partial ^{2}p_{k,\omega }(x)}{\partial x_{n}\partial x_{k-1}}\\ \frac{\partial ^{2}p_{k,\omega }(x)}{\partial x_{1}\partial x_{k+1}} &{} \cdots &{} \frac{\partial ^{2}p_{k,\omega }(x)}{\partial x_{k-1}\partial x_{k+1}} &{} \frac{\partial ^{2}p_{k,\omega }(x)}{\partial x_{k+1}^{2}} &{} \cdots &{} \frac{\partial ^{2}p_{k,\omega }(x)}{\partial x_{n}\partial x_{k+1}}\\ \vdots &{} &{} \vdots &{} \vdots &{} \ddots &{} \vdots \\ \frac{\partial ^{2}p_{k,\omega }(x)}{\partial x_{1}\partial x_{n}} &{} \cdots &{} \frac{\partial ^{2}p_{k,\omega }(x)}{\partial x_{k-1}\partial x_{n}} &{} \frac{\partial ^{2}p_{k,\omega }(x)}{\partial x_{k+1}\partial x_{n}} &{} \cdots &{} \frac{\partial ^{2}p_{k,\omega }(x)}{\partial x_{n}^{2}} \end{array} \right) \text {,} \end{aligned}$$

is positive semi-definite at *x*. In other words, $z_{-k}^{T}H_{k,\omega }(x)z_{-k}\ge 0$ for any

41 $$\begin{aligned} z_{-k}=(z_{1},\ldots ,z_{k-1},z_{k+1},\ldots ,z_{n})^{T}\in {\mathbb {R}} ^{n-1}. \end{aligned}$$

Consider now the matrix

42 $$\begin{aligned} H_{k,\omega }^{0}(x)=\left( \begin{array} [c]{ccccccc} \frac{\partial ^{2}p_{k,\omega }(x)}{\partial x_{1}^{2}} &{} \cdots &{} \frac{\partial ^{2}p_{k,\omega }(x)}{\partial x_{k-1}\partial x_{1}} &{} 0 &{} \frac{\partial ^{2}p_{k,\omega }(x)}{\partial x_{k+1}\partial x_{1}} &{} \cdots &{} \frac{\partial ^{2}p_{k,\omega }(x)}{\partial x_{n}\partial x_{1}}\\ \vdots &{} \ddots &{} \vdots &{} \vdots &{} \vdots &{} &{} \vdots \\ \frac{\partial ^{2}p_{k,\omega }(x)}{\partial x_{1}\partial x_{k-1}} &{} \cdots &{} \frac{\partial ^{2}p_{k,\omega }(x)}{\partial x_{k-1}^{2}} &{} 0 &{} \frac{\partial ^{2}p_{k,\omega }(x)}{\partial x_{k+1}\partial x_{k-1}} &{} \cdots &{} \frac{\partial ^{2}p_{k,\omega }(x)}{\partial x_{n}\partial x_{k-1}}\\ 0 &{} \cdots &{} 0 &{} 0 &{} 0 &{} \cdots &{} 0\\ \frac{\partial ^{2}p_{k,\omega }(x)}{\partial x_{1}\partial x_{k+1}} &{} \cdots &{} \frac{\partial ^{2}p_{k,\omega }(x)}{\partial x_{k-1}\partial x_{k+1}} &{} 0 &{} \frac{\partial ^{2}p_{k,\omega }(x)}{\partial x_{k+1}^{2}} &{} \cdots &{} \frac{\partial ^{2}p_{k,\omega }(x)}{\partial x_{n}\partial x_{k+1}}\\ \vdots &{} &{} \vdots &{} \vdots &{} \vdots &{} \ddots &{} \vdots \\ \frac{\partial ^{2}p_{k,\omega }(x)}{\partial x_{1}\partial x_{n}} &{} \cdots &{} \frac{\partial ^{2}p_{k,\omega }(x)}{\partial x_{k-1}\partial x_{n}} &{} 0 &{} \frac{\partial ^{2}p_{k,\omega }(x)}{\partial x_{k+1}\partial x_{n}} &{} \cdots &{} \frac{\partial ^{2}p_{k,\omega }(x)}{\partial x_{n}^{2}} \end{array} \right) \text {.} \end{aligned}$$

It is straightforward to check that $z^{T}H_{k,\omega }^{0}(x)z=z_{-k} ^{T}H_{k,\omega }(x)z_{-k}\ge 0$ for any $z=(z_{1},\ldots ,z_{n})^{T}\in {\mathbb {R}} ^{n}$. Thus, the matrix $H_{k,\omega }^{0}(x)$ is likewise positive semi-definite. Summing now over $k=1,\ldots ,n$, we obtain that

43 $$\begin{aligned} \sum _{k=1}^{n}H_{k,\omega }^{0}(x)=\left( \begin{array} [c]{cccc} {\sum }_{k\ne 1}\frac{\partial ^{2}p_{k,\omega }(x)}{\partial x_{1}^{2}} &{} {\sum }_{k\ne 2,1}\frac{\partial ^{2}p_{k,\omega }(x)}{\partial x_{2}\partial x_{1}} &{} \cdots &{} \sum _{k\ne n,1}\frac{\partial ^{2}p_{k,\omega }(x)}{\partial x_{n}\partial x_{1}}\\ {\sum }_{k\ne 1,2}\frac{\partial ^{2}p_{k,\omega }(x)}{\partial x_{1}\partial x_{2}} &{} {\sum }_{k\ne 2}\frac{\partial ^{2}p_{k,\omega }(x)}{\partial x_{2}^{2}} &{} &{} \vdots \\ \vdots &{} &{} \ddots &{} \vdots \\ {\sum }_{k\ne 1,n}\frac{\partial ^{2}p_{k,\omega }(x)}{\partial x_{1}\partial x_{n}} &{} \cdots &{} \cdots &{} {\sum }_{k\ne n}\frac{\partial ^{2}p_{k,\omega } (x)}{\partial x_{n}^{2}} \end{array} \right) \text {.} \end{aligned}$$

Since $x\ne 0$, we have $\sum _{k=1}^{n}p_{k,\omega }(x)=1$ and, consequently, $\sum _{k=1}^{n}\frac{\partial ^{2}p_{k,\omega }(x)}{\partial x_{i}\partial x_{j}}=0$ for arbitrary $i,j\in N$. Hence,

44 $$\begin{aligned}&-\sum _{k=1}^{n}H_{k,\omega }^{0}(x)\nonumber \\&\quad =\left( \begin{array} [c]{cccc} \frac{\partial ^{2}p_{1,\omega }(x)}{\partial x_{1}^{2}} &{} \frac{\partial ^{2}p_{1,\omega }(x)}{\partial x_{2}\partial x_{1}}+\frac{\partial ^{2}p_{2,\omega }(x)}{\partial x_{2}\partial x_{1}} &{} \cdots &{} \frac{\partial ^{2}p_{1,\omega }(x)}{\partial x_{n}\partial x_{1}}+\frac{\partial ^{2}p_{n,\omega }(x)}{\partial x_{n}\partial x_{1}}\\ \frac{\partial ^{2}p_{1,\omega }(x)}{\partial x_{1}\partial x_{2}} +\frac{\partial ^{2}p_{2,\omega }(x)}{\partial x_{1}\partial x_{2}} &{} \frac{\partial ^{2}p_{2,\omega }(x)}{\partial x_{2}^{2}} &{} &{} \vdots \\ \vdots &{} &{} \ddots &{} \vdots \\ \frac{\partial ^{2}p_{1,\omega }(x)}{\partial x_{1}\partial x_{n}} +\frac{\partial ^{2}p_{n,\omega }(x)}{\partial x_{1}\partial x_{n}} &{} \cdots &{} \cdots &{} \frac{\partial ^{2}p_{n,\omega }(x)}{\partial x_{n}^{2}} \end{array} \right) \end{aligned}$$

is negative semi-definite. Moreover, using that $p_{i,\omega }(\cdot )$ is of the logit form, it is straightforward to check that $\frac{\partial ^{2}p_{i,\omega }(x)}{\partial x_{i}\partial x_{j}}=\frac{\partial ^{2}p_{i,\omega }(x)}{\partial x_{j}\partial x_{i}}$ holds for any $i,j\in N$ such that $j\ne i$. Thus, $J_{p,\omega }(x)+J_{p,\omega }(x)^{T}=M_{\omega }(x)-\sum _{k=1}^{n}H_{k,\omega }^{0}(x)$, and the claim follows. Let now $x\in {\mathbb {R}} _{+}^{n}$ possess two or more nonzero components. Then, $x_{-i}\in {\mathbb {R}} _{+}^{n-1}\backslash \{0\}$ for all $i\in N$. In this case, therefore, $M_{\omega }(x)$ is negative definite, and so is $J_{p,\omega }(x)+J_{p,\omega }(x)^{T}$.

$$\square $$

The next lemma establishes an important convexity property of logit contests.

### Lemma A.5

For any $i\in N$, $\omega \in \Omega$, and $x_{i}\in {\mathbb {R}} _{+}$ such that $f_{i,\omega }(x_{i})>0$, the mapping $x_{-i}\mapsto p_{i,\omega }(x_{i},x_{-i})$ is convex on ${\mathbb {R}} _{+}^{n-1}$.

### Proof

Note first that the mapping $x_{-i}\mapsto \sum _{j\ne i}f_{j,\omega }(x_{j})$ is concave on ${\mathbb {R}} _{+}^{n-1}$. Indeed, by Assumption (A), its Hessian is a diagonal matrix with weakly negative entries. Further, for $f_{i,\omega }(x_{i})>0$ fixed, the mapping $Y\mapsto \frac{f_{i,\omega }(x_{i})}{f_{i,\omega }(x_{i})+Y}$ is convex and decreasing on ${\mathbb {R}} _{++}$. The claim follows therefore from Rockafellar (1970, Theorem 5.1). $\square$

The lemma below recalls two simple matrix-theoretic facts.

### Lemma A.6

Let $J\in {\mathbb {R}} ^{n\times n}$. If $J+J^{T}$ is negative semi-definite, then $z^{T}Jz\le 0$ for any $z\in {\mathbb {R}} ^{n}$. If $J+J^{T}$ is even negative definite, then $z^{T}Jz<0$ for any $z\in {\mathbb {R}} ^{n}\backslash \{0\}$.

### Proof

Clearly, $z^{T}Jz=((z^{T}J)z)^{T}=z^{T}(z^{T} J)^{T}=z^{T}J^{T}(z^{T})^{T}=z^{T}J^{T}z$. Hence, $2z^{T}Jz=z^{T}Jz+z^{T} J^{T}z=z^{T}(Jz+J^{T}z)=z^{T}(J+J^{T})z$. The assertions follow. $\square$

The following lemma is crucial for obtaining the strict inequality in the proof of Theorem 2.

### Lemma A.7

Let $$\beta ^{*}\ne \beta ^{**}$$ be two PSNE. Then, there is a state $$\omega _{0}\in \Omega $$ such that the following two conditions hold true:

(i) for any $s\in (0,1)$, the profile of bids $\beta ^{s}(t(\omega _{0}))$ has at least two nonzero components;

(ii) $$z(\omega _{0})=\beta ^{*}(t(\omega _{0}))-\beta ^{**}(t(\omega _{0}))\ne 0$$.

### Proof

Select some $$i\in N$$ and $$\theta _{i}\in \Theta _{i}$$ such that $$\beta _{i}^{*}(\theta _{i})\ne \beta _{i}^{**}(\theta _{i})$$. Then, clearly, $$x_{i}^{\max }(\theta _{i})>0$$. Moreover, by strict quasiconcavity of type $$\theta _{i}$$’s equilibrium payoff function, there must be a state $$\omega _{0}\in {\mathcal {P}}_{i}(\theta _{i})$$ such that $$\beta _{-i}^{*}(t_{-i}(\omega _{0}))\ne \beta _{-i}^{**}(t_{-i}(\omega _{0} ))$$. Therefore, $$\beta _{-i}^{s}(t_{-i}(\omega _{0}))\ne 0$$ for any $$s\in (0,1)$$. But from $$\beta _{i}^{*}(\theta _{i})\ne \beta _{i}^{**}(\theta _{i})$$, it similarly follows that $$\beta _{i}^{s}(t_{i}(\omega _{0}))\ne 0$$ for any $$s\in (0,1)$$. Thus, the vector $$\beta ^{s}(t(\omega _{0}))$$ indeed has at least two nonzero components, for any $$s\in (0,1)$$. Moreover, $$z_{i}(\omega _{0})=\beta _{i}^{*}(t_{i}(\omega _{0}))-\beta _{i}^{**}(t_{i} (\omega _{0}))\ne 0$$, so that $$z(\omega _{0})\ne 0$$. This proves the lemma. $$\square $$

### Proof of Theorem 3

*(Existence) *For any $i\in N$, let

45 $$\begin{aligned} \Pi _{i}^{\text {ex ante}}(\beta )=\sum \nolimits _{\omega \in \Omega }q(\omega )\Pi _{i,\omega }(\beta (t(\omega ))) \end{aligned}$$

denote player *i*’s ex ante expected payoff from the pure-strategy profile $\beta \in B$. By Lemma A.8, the *n*-player game defined through payoff functions (45) is symmetric. Choose now a sequence $\{\varepsilon _{m}\}_{m=1}^{\infty }$ in $\mathbb {(}0,{\overline{\varepsilon }})$ with $\lim _{m\rightarrow \infty }\varepsilon _{m}=0$. Since $x_{\pi (i)}^{\max } (\cdot )=x_{i}^{\max }(\cdot )$ for any $i\in N$ and any permutation $\pi$, also ${\mathcal {C}}(\varepsilon _{m})$, defined in the proof of Theorem 1, is a symmetric *n*-player game. Hence, by a well-known variant of the Nikaidô–Isoda theorem (Moulin 1986, pp. 115–116; see also Becker and Damianov 2006), there exists a symmetric PSNE $\beta ^{m}$ in the *n*-player game ${\mathcal {C}}(\varepsilon _{m})$, for any *m*. We may assume w.l.o.g. that the sequence $\left\{ \beta ^{m}\right\} _{m=1}^{\infty }$ converges to some limit $\beta ^{*}\in B$. Clearly, $\beta ^{*}$ is a symmetric strategy profile in ${\mathcal {C}}$. Take any $i\in N$ and $\theta _{i}\in \Theta _{i}$ such that $x_{i}^{\max }(\theta _{i})>0$, and let $\omega \in {\mathcal {P}}_{i} (\theta _{i})$. As in the proof of Theorem 1, one checks that $\Pi _{i,\omega }(\cdot )$ is continuous at $\beta ^{*}(t(\omega ))$ if either condition (i) or condition (ii) of Assumption (B’) holds. To prove the existence part, it therefore suffices to show that, also under condition (iii) of Assumption (B’), $\Pi _{i,\omega }(\cdot )$ is continuous at $\beta ^{*}(t(\omega ))$. For this, let $\omega ^{\#}\in {\mathcal {P}}_{i}(\theta _{i})$ be activity-inducing for player *i*. Then, (ı) the contest is discontinuous in $\omega ^{\#}$, (ıı) there exists $j\in N\backslash \{i\}$ such that $x_{j}^{\max }(t_{j}(\omega ^{\#}))>0$, and (ııı) for any $k\in N\backslash \{i\}$ such that $x_{k}^{\max }(t_{k}(\omega ^{\#}))>0$, it holds that $t_{k}(\omega ^{\#} )=t_{i}(\omega ^{\#})$. Now, since $\omega ^{\#}\in {\mathcal {P}}_{i}(\theta _{i})$, we have $t_{i}(\omega ^{\#})=t_{i}(\omega )=\theta _{i}$. Hence, $x_{i}^{\max }(t_{i}(\omega ^{\#}))>0$. In view of (ı) and (ıı), Lemma A.1 implies that $\beta ^{*}(t(\omega ))\ne 0$. Moreover, from (ııı) and the fact that $\beta ^{*}$ is a symmetric strategy profile, it follows that, for any $k\in N\backslash \{i\}$ with $x_{k}^{\max }(t_{k}(\omega ^{\#}))>0$, we have $\beta _{k}^{*}(t_{k}(\omega ^{\#}))=\beta _{i}^{*}(t_{i}(\omega ^{\#}))$. In particular, $\beta _{i}^{*}(t_{i}(\omega ^{\#}))>0$. But since $t_{i}(\omega )=t_{i}(\omega ^{\#})$, we find that $\beta _{i}^{*} (t_{i}(\omega ))>0$, so that $\Pi _{i,\omega }(\cdot )$ is indeed continuous at $\beta ^{*}(t(\omega ))$. *(Uniqueness)* Immediate from Theorem 2. $\square$

Our final lemma says that the payoff functions specified in (45) define a symmetric *n*-player game.

### Lemma A.8

For any $i\in N$, any $\beta =(\beta _{1},\ldots ,\beta _{n})\in B$, and any permutation $\pi :N\rightarrow N$, it holds that $\Pi _{i}^{\text {ex ante}} (\beta _{\pi (1)},\ldots ,\beta _{\pi (n)})=\Pi _{\pi (i)}^{\text {ex ante}}(\beta _{1},\ldots ,\beta _{n})$.

### Proof

Take any $i\in N$, any $\beta \in B$, and any permutation $\pi$. Consider an arbitrary state $\omega \in \Omega$ and the corresponding vector of bid realizations $x=\beta (t(\omega ))\in {\mathbb {R}} _{+}^{n}$. Since $f_{j,\omega ^{\pi }}(\cdot )=f_{\pi (j),\omega }(\cdot )$ for any $j\in N$, we have

46 $$\begin{aligned} \sum _{j=1}^{n}f_{j,\omega ^{\pi }}(x_{\pi (j)})=\sum _{j=1}^{n}f_{\pi (j),\omega }(x_{\pi (j)})\text {.} \end{aligned}$$

If the left-hand side of (46) is positive, then

47 $$\begin{aligned} p_{i,\omega ^{\pi }}(x^{\pi })=\dfrac{f_{i,\omega ^{\pi }}(x_{\pi (i)})}{\textstyle \sum _{j=1}^{n}f_{j,\omega ^{\pi }}(x_{\pi (j)})}=\dfrac{f_{\pi (i),\omega }(x_{\pi (i)})}{\textstyle \sum _{j=1}^{n}f_{\pi (j),\omega }(x_{\pi (j)})}=p_{\pi (i),\omega }(x)\text {.} \end{aligned}$$

If, however, the left-hand side of (46) vanishes, then likewise $p_{i,\omega ^{\pi }}(x^{\pi })=p_{i,\omega ^{\pi }}^{0}=p_{\pi (i),\omega } ^{0}=p_{\pi (i),\omega }(x)$. Therefore,

48 $$\begin{aligned} \Pi _{i,\omega ^{\pi }}(x^{\pi })&=p_{i,\omega ^{\pi }}(x^{\pi })v_{i} (\omega ^{\pi })-c_{i,\omega ^{\pi }}(x_{\pi (i)}) \end{aligned}$$

49 $$\begin{aligned}&=p_{\pi (i),\omega }(x)v_{\pi (i)}(\omega )-c_{\pi (i),\omega }(x_{\pi (i)}) \end{aligned}$$

50 $$\begin{aligned}&=\Pi _{\pi (i),\omega }(x)\text {.} \end{aligned}$$

It is now straightforward to check that

51 $$\begin{aligned}&\Pi _{i}^{\text {ex ante}}(\beta _{\pi (1)},\ldots ,\beta _{\pi (n)})\underset{(45)}{=}\sum \limits _{\omega \in \Omega }q(\omega )\Pi _{i,\omega }(\beta _{\pi (1)}(t_{1}(\omega )),\ldots ,\beta _{\pi (n)}(t_{n} (\omega ))) \end{aligned}$$

52 $$\begin{aligned}&\underset{\pi \text { one-to-one}}{=}\sum \limits _{\omega \in \Omega } q(\omega ^{\pi })\Pi _{i,\omega ^{\pi }}(\beta _{\pi (1)}(t_{1}(\omega ^{\pi })),\ldots ,\beta _{\pi (n)}(t_{n}(\omega ^{\pi }))) \end{aligned}$$

53 $$\begin{aligned}&\underset{t_{j}(\omega ^{\pi })=t_{\pi (j)}(\omega )}{=}\sum \limits _{\omega \in \Omega }q(\omega ^{\pi })\Pi _{i,\omega ^{\pi }}(\beta _{\pi (1)}(t_{\pi (1)} (\omega )),\ldots ,\beta _{\pi (n)}(t_{\pi (n)}(\omega ))) \end{aligned}$$

54 $$\begin{aligned}&\underset{(48-50)}{=}\sum \limits _{\omega \in \Omega }q(\omega ^{\pi })\Pi _{\pi (i),\omega }(\beta _{1}(t_{1}(\omega )),\ldots ,\beta _{n}(t_{n}(\omega ))) \end{aligned}$$

55 $$\begin{aligned}&\underset{q(\omega ^{\pi })=q(\omega )}{=}\sum \limits _{\omega \in \Omega }q(\omega )\Pi _{\pi (i),\omega }(\beta _{1}(t_{1}(\omega )),\ldots ,\beta _{n} (t_{n}(\omega ))) \end{aligned}$$

56 $$\begin{aligned}&\underset{(45)}{=} {\Pi _{\pi (i)}^{\text {ex ante}}}(\beta _{1},\ldots \beta _{n}), \end{aligned}$$

which proves the claim. $\square$

## Footnotes

1 See Vojnović (2016) for an introduction to the theory of contests.

2
Einy et al. (2015) independently proved existence in a framework similar to ours. In fact, some of the steps in their initial proof (Einy et al. 2013) happen to correspond to steps in our proof. However, Einy et al. (2015) do not allow for budget constraints. More importantly, they do not offer general conditions for uniqueness. See also the discussion of the related literature in Sect. 2.

3 For the same reasons, Ui’s (2016, Cor. 6) recent extension of Rosen’s theorem actually fails to apply to Tullock’s rent-seeking game.

4 Franke and Öztürk (2015, p. 105) have applied Goodman’s result to networks of two-player contests with continuous technologies and complete information. However, as they emphasize, their proof crucially relies on the additive separability of payoffs across opponents, which is, in general, not satisfied in contests with incomplete information.

5 For example, Leininger (1991) emphasized the role of research budgets for the persistence of monopoly. In the literature on lobbying caps (Che and Gale 1998), the introduction of spending limits reduces preemption and may thereby increase the expected revenue of a politician. Konrad (2012) studied the formation of alliances whose members share private information on budgets.

6 To prove uniqueness, Wasser (2013a) employs techniques due to Mason and Valentinyi (2010).

7 To deal with PSNE existence in isolation, one may alternatively assume that strategy spaces are discrete (see, e.g., Singh and Wittman 1988). However, that assumption tends to make uniqueness less likely, even under complete information.

8
Yamazaki (2008) used a similar argument.

9 State-contingent impact functions arise, for example, when there is incomplete information about abilities (Baik and Shogren 1995; Clark 1997; Epstein and Mealem 2013) or about technological parameters (Grossmann 2014; Heijnen and Schoonbeek 2019).

10 The expectations exist provided that cost functions are bounded on compact intervals. In particular, this is so under Assumption (A), which will be introduced in the next section.

11 As far as existence is concerned, twice differentiability of $f_{i,\omega }$ at the zero bid could actually be replaced by continuity at the zero bid without affecting the conclusions. However, twice differentiability of $f_{i,\omega }$ at the zero bid will be crucial in our proof of uniqueness, which is why we impose this assumption right from the start.

12 The extension to the case where $f_{i,\omega }(0)$ may be positive is straightforward.

13 As will be illustrated below, this problem does not go away when budgets are private information.

14 In fact, as will be discussed at the end of the next section, there is no MSNE either.

15 For instance, when several firms $i=1,\ldots ,n$ exploit a natural resource, the common factor $v(\cdot )$ might correspond to the total yield, while $\kappa _{i}(t_{i}(\cdot ))$ might correspond to firm *i*’s profitability. Note also that Assumption (C) holds trivially under complete information.

16 Indeed, from Assumption (C), $v_{1}(\omega )=v(\omega )\cdot \kappa _{1}(\theta _{1})$ and $v_{2}(\omega )=v(\omega )\cdot \kappa _{2}(\theta _{2})$. But if $\theta _{1}=\theta _{2}$, then $v_{1}(\omega )=(1+\alpha )\theta _{1}=(1+\alpha )\theta _{2}=v_{2}(\omega )$. Hence, $\kappa _{1}(\theta _{1})=\kappa _{2}(\theta _{2})$, as claimed.

17 The figure shows the gradient field for a two-player Tullock contest with heterogeneous valuations and positive budgets. For reasons of dimensionality, the example assumes complete information. Moreover, given the singularity at the origin, the gradient field has been normalized.

18 In fact, Assumption (C) is not needed for this conclusion.

19 If the contest is continuous in all states of the world, then the second part of Theorem 2 may be derived alternatively by combining Lemma A.5 in the Appendix with Ui (2008, Cor. 6). However, that alternative proof does not go through under the more general assumptions of Theorem 2.

20 In particular, condition (ııı) implies that players *i* and *j* are of the same type.

21 Depending on the setup, the set of potential entrants may be finite (Münster 2006; Lagerlöf 2007; Lim and Matros 2009; Fu et al. 2011) or infinite (Myerson and Wärneryd 2006; Kahana and Klunover 2015).

22 Instead, he shows that any symmetric PSNE, if it exists, satisfies a system of two equations (which, in principle, might have several solutions or no solution, even if players’ objective functions are globally concave).

23 However, the general analysis of uniqueness for contests with interdependent valuations remains an open issue.

24 Very recently, Xu et al. (2019) have applied the methods of the present paper to networks of contests.
